# Electrocatalytic CO_2_ Reduction to Alcohols: Progress and Perspectives

**DOI:** 10.1002/smsc.202400129

**Published:** 2024-06-11

**Authors:** Ying Long, Zhijie Chen, Lan Wu, Xiaoqing Liu, Ya‐Nan Hou, Sergio Vernuccio, Wei Wei, Wai‐Yeung Wong, Bing‐Jie Ni

**Affiliations:** ^1^ Centre for Technology in Water and Wastewater School of Civil and Environmental Engineering University of Technology Sydney Sydney NSW 2007 Australia; ^2^ School of Civil and Environmental Engineering University of New South Wales Sydney NSW 2052 Australia; ^3^ Department of Civil and Environmental Engineering The Hong Kong Polytechnic University Kowloon Hong Kong P. R. China; ^4^ Tianjin Key Laboratory of Aquatic Science and Technology School of Environmental and Municipal Engineering Tianjin Chengjian University Jinjing Road 26 Tianjin 300384 China; ^5^ Department of Chemical and Biological Engineering University of Sheffield Sheffield S10 2TN UK; ^6^ Department of Applied Biology and Chemical Technology and Research Institute for Smart Energy The Hong Kong Polytechnic University Hung Hom, Kowloon Hong Kong P. R. China

**Keywords:** alcohols, carbon dioxide reductions, carbon valorizations, electrocatalysts, electrolyzers

## Abstract

Utilizing renewable electricity for the electrocatalytic conversion of CO_2_ into alcohols represents a promising avenue for generating value‐added fuels and achieving carbon neutrality. Recently, there has been growing scientific interest in achieving high‐efficiency conversion of CO_2_ to alcohols, with significant advancements made in mechanism understanding, reactor design, catalyst development, and more. Herein, a thorough examination of the latest advances in electrocatalytic CO_2_ reduction reaction (CO_2_RR) to alcohols is provided. General mechanisms and pathways of electrocatalytic conversion of CO_2_‐to‐alcohols are systematically illustrated. Subsequently, electrolyzer configurations, electrolytes, and electrocatalysts employed in CO_2_RR are summarized. After that, critical operating parameters (e.g., reaction pressure, temperature, and pH) that would significantly influence the CO_2_RR process are also analyzed. Finally, the review addresses challenges and offers perspectives in this field to guide future studies aimed at advancing CO_2_‐to‐alcohols conversion technologies.

## Graphical abstract

This article undertakes a comprehensive scrutiny of the reaction mechanisms and pathways of electrocatalytic CO_2_ reduction (Co_2_RR) to alcohol production. Electrolyzers, electrolytes, and electrocatalysts employed in CO_2_‐to‐alcohol conversion are thoroughly analyzed. The effects of various reaction parameters on the CO_2_RR process are discussed. Furthermore, challenges in this field are outlined to guide future research on the valorization of CO_2_.

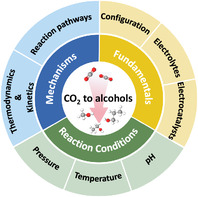

## 1 Introduction

The exacerbating expansion of the global economy, coupled with the excessive utilization of fossil fuels, is intensifying the gravity of both the energy crisis and global warming.^[^ ^1^, ^2^ ^]^ Addressing this challenge requires considerable attention to the electrocatalytic CO_2_ reduction reaction (CO_2_RR) driven by renewable energy sources.^[^ ^3^ ^]^ This area has garnered significant research interest due to its potential as an environmentally friendly solution to issues arising from escalating CO_2_ emissions. By leveraging clean energy, electrocatalytic CO_2_RR not only contributes to mitigating environmental problems but also yields valuable, high‐value‐added products.^[^ ^4^, ^5^, ^6^, ^7^, ^8^, ^9^ ^]^ Various compounds (e.g., CO, CH_4_, HCOOH, CH_3_OH, C_2_H_4_, CH_3_COOH, C_2_H_5_OH, C_3_H_7_OH) can be generated through the electrocatalytic conversion of CO_2_, contingent upon the number of electrons transferred.^[^ ^10^ ^]^ Among these, alcohols stand out as one of the most prevalent and utilitarian classes of CO_2_RR products.^[^ ^11^, ^12^ ^]^ In this regard, alcohols have widespread applications as an additive in automotive gasoline and play a pivotal role in the synthesis of diverse organic compounds.^[^ ^13^ ^]^ Additionally, alcohols serve as crucial liquid fuels, valued for their high energy densities and ease of transport and storage.^[^ ^14^ ^]^ Gas hydrogenation and crop fermentation are the traditional methods of alcohol production. However, their application is restricted due to energy‐intensive demands and concerns related to biodiversity and food security.^[^ ^15^ ^]^ In contrast, CO_2_RR offers a more sustainable alternative by converting CO_2_ into alcohols under ambient conditions. This approach simultaneously contributes to a reduction in the carbon footprint associated with green alcohol production, presenting a promising avenue for addressing the challenges posed by conventional methods.

Considerable efforts have been exerted to augment both the yield and selectivity of electrocatalytic alcohol production processes, such as reactor design, electrolyte selection, and electrocatalyst optimization. Initially, advancements such as H‐type electrolyzers, flow cells, and membrane electrode assembly (MEA) reactors were developed aimed at improving the reaction efficiency. Additionally, tandem electrolysis systems have emerged as a promising approach toward enhanced alcohol production rates. Electrolytes are important components in CO_2_RR, and various mediums, with both aqueous solution (e.g., KOH, KHCO_3_, KCl) and ionic liquids (ILs) (e.g., [Bmim]BF_4_) demonstrating potential for alcohol production. Cu‐based materials stand out as the predominant electrocatalysts under investigation. Diverse strategies have been implemented to tailor catalyst properties, encompassing the construction of single‐atom catalysts (SACs), optimization of chemical composition, control of nanostructure, doping with heteroatom, and construction of heterostructure.^[^ ^16^, ^17^ ^]^ For example, carbon‐supported copper catalysts^[^ ^18^ ^]^ and ultrahigh‐density Cu SACs loaded on thin‐walled N‐doped carbon nanotubes (TWN)^[^ ^19^ ^]^ have been utilized to produce C_2_H_5_OH with high selectivity. Additionally, fine‐tuned Cu_2_O with nanostructure catalysts for alcohol production have been extensively investigated.^[^ ^20^, ^21^ ^]^ Furthermore, metal‐carbon support‐based,^[^ ^22^, ^23^, ^24^ ^]^ multimetallic composition,^[^ ^25^, ^26^ ^]^ and metal oxide‐based heterostructure catalysts^[^ ^27^, ^28^ ^]^ have also demonstrated excellent performance in electrocatalytic CO_2_RR.

The impact of electrolysis protocols on reaction efficiency is a significant consideration in current research. To date, extensive investigations have been conducted into the pivotal factors of pressure, temperature, and electrolyte pH value. For instance, Li et al.^[^ ^29^ ^]^ found that the cathode electrochemically reduces CO_2_ to almost pure formate at pressure ≥45 atm CO_2_ in a bicarbonate catholyte. Analogous findings have been corroborated in other studies.^[^ ^30^, ^31^ ^]^ Lin and colleagues noticed that the faradaic efficiencies (FE) of CO over Fe–N–C and Ni–N–C catalysts at high overpotentials show an opposite trend as the temperature increases within a certain range.^[^ ^32^ ^]^ It has been also reported that the selectivity of oxide‐derived copper electrodes in electrocatalytic CO_2_RR is significantly influenced by the pH level near the electrode surface.^[^ ^33^ ^]^ These results largely promote the understanding of the CO_2_RR systems and offer insights for regulating the CO_2_RR pathway to attain target products. While numerous commendable review papers on the electrochemical CO_2_RR have been published, the majority primarily focus on describing the electrocatalytic aspects of CO_2_ reduction^[^ ^34^, ^35^, ^36^, ^37^ ^]^ and the strategies for optimizing catalysts.^[^ ^38^, ^39^, ^40^, ^41^, ^42^ ^]^ To our knowledge, a systematic and comprehensive summary of CO_2_RR to alcohols remains conspicuously absent in the existing literature.

This review focuses on the latest developments in the realm of CO_2_RR to produce alcohol products. General mechanisms and pathways of CO_2_‐to‐alcohols have been especially discussed first. And then, we introduce the fundamentals of electrocatalytic CO_2_RR to alcohols in the following section. Subsequently, the impact of important experimental parameters, such as operating pressure, temperature, and pH is critically reviewed as these factors can guide product selectivity by controlling the reaction pathways. Finally, a comprehensive summary of the challenges encountered in the field is provided, followed by a critical discussion on future perspectives. This review will inspire future studies to advance CO_2_RR techniques toward the realization of high‐efficiency sustainable fuel production.

## 2 Mechanisms of CO_2_RR to Alcohols

CO_2_RR can generate a wide array of products, ranging from single‐carbon products to high‐value multi‐carbon products through various reaction pathways and mechanisms that involve complex proton‐associated multielectron transfer processes.^[^ ^43^ ^]^ Hence, it is vital to control the reaction pathways of CO_2_RR and elucidate the thermodynamics and kinetics mechanisms that enhance the selectivity toward desired products. Lately, rapid advancements in in situ X‐ray diffraction, surface‐enhanced infrared adsorption spectroscopy, Raman spectroscopy, X‐ray absorption spectroscopy (XAS), nuclear magnetic resonance (NMR), and density functional theory (DFT) calculations offered effective tools to address these challenges.^[^ ^44^ ^]^

### 2.1 Thermodynamics and Kinetics of CO_2_RR

The primary products of the CO_2_RR vary according to the number of electrons transferred (e.g., 2e^−^: HCOOH and CO, 6e^−^: CH_3_OH, 8e^−^: CH_4_ and CH_3_COOH, 12e^−^: C_2_H_4_ and C_2_H_5_OH, 18e^−^: C_3_H_7_OH).^[^ ^45^, ^46^ ^]^ The thermodynamics of CO_2_RR are primarily governed by the standard Gibbs free energies associated with the reactants and products involved. The half‐electrochemical equilibrium potentials for CO_2_ reduction are displayed in Reactions 1–6 (V versus reversible hydrogen electrode (RHE)).^[^ ^40^ ^]^

#### 2.1.1 Protonation Process

(1) $${\text{CO}}_{2}{\text{+ 6H}}^{+}{\text{+ 6e}}^{‐}\to \;{\text{CH}}_{3}\text{OH}\;+\;H_{2}\text{O }E_{\text{redox}}^{0}\;\text{= 0}\text{.016 V}$$

(2) $${\text{2CO}}_{2}{\text{+ 12H}}^{+}{\text{+ 12e}}^{‐}\to {\text{ C}}_{2}H_{5}{\text{OH + 3H}}_{2}O\;\;E_{\text{redox}}^{0}\;=\;0\text{.084 V}$$

(3) $${\text{3CO}}_{2}{\text{+ 18H}}^{+}{\text{+ 18e}}^{‐}\to {\text{ C}}_{3}H_{7}{\text{OH + 5H}}_{2}O\;\;E_{\text{redox}}^{0}\text{ = 0}\text{.095 V}$$

#### 2.1.2 Hydration Reaction Process

(4) $${\text{CO}}_{2}\;{\text{+ 5H}}_{2}O\;{\text{+ 6e}}^{-}\;\to \;{\text{CH}}_{3}\text{OH}\;{\text{+ 6OH}}^{-}\;\;E_{\text{redox}}^{0}\;=\;\text{−0}\text{.812 V}$$

(5) $${\text{2CO}}_{2}\;{\text{+ 9H}}_{2}O\;{\text{+ 12e}}^{-}\;\to \;C_{2}H_{5}\text{OH}\;+\;{\text{12OH}}^{-}\;\;E_{\text{redox}}^{0}\;\text{= −0}\text{.744 V}$$

(6) $${\text{3CO}}_{2}\;{\text{+ 13H}}_{2}O\;+\;{\text{18e}}^{-}\;\to \;C_{3}H_{7}\text{OH}\;{\text{+ 18OH}}^{-}\;\;E_{\text{redox}}^{0}\;\text{= −0}\text{.733 V}$$

The initial electron transfer step, commonly accepted as the rate‐limiting step (RLS), involves the direct reduction of CO_2_ (CO_2_ + e^−^ → CO_2_^•−^).^[^ ^47^ ^]^ The transformation of CO_2_ into CO_2_^•−^ radical anion demands a considerable input of energy due to the thermodynamic stability of the CO_2_ molecule. Thus, a markedly elevated overpotential is essential to initiate the process and break the C=O bond. It is noteworthy that the hydrogen evolution reaction (HER) always accompanies the CO_2_RR in aqueous electrolytes as the thermodynamic reaction potentials of the protonation process of various products are all close to 0 V versus RHE.^[^ ^48^ ^]^ It is important to emphasize that the conversion of CO_2_RR to hydrocarbons or alcohols is a more intricate and less reactive process. Despite the thermodynamic potentials of CO_2_RR to hydrocarbons or alcohols being more favorable than those of H_2_, CO, and HCOOH, this reaction poses greater complexity. Beyond thermodynamic consideration, the success of CO_2_RR is also contingent on kinetic factors such as the presence of protons in the electrolyte, the formation of *CO intermediates, and the coverage on the catalyst surface.^[^ ^49^ ^]^

The performance of the CO_2_RR is significantly influenced by reaction kinetics, particularly the barrier hindering the high electron approach to the external surface sphere. This barrier arises from the distinct structures of CO_2_ and its radical anion. The resulting potential is much more negative than what is needed for the generation of most CO_2_ reduction products, leading to tremendous overpotentials during the reaction. In the thermodynamically favored protonation process, C_2_H_5_OH and C_3_H_7_OH exhibit more positive standard potentials than CH_3_OH. This indicates the thermodynamic feasibility of producing multicarbon alcohols. However, the rate and selectivity of C_2_ and higher hydrocarbon production are constrained by a high kinetic energy barrier. Hydrogenation of bound C_1_ species is kinetically favorable compared to the chemical C—C coupling reaction.^[^ ^49^, ^50^ ^]^ This implies that the generation of CH_3_OH is much easier than that of C_2+_ alcohols and typically does not require extremely high overpotentials. Aside from the high kinetic barrier for CO_2_ activation, the side reaction of the HER also hampers CO_2_RR kinetics.^[^ ^51^ ^]^ Consequently, achieving potentials more negative than the thermodynamic potentials of the different products becomes necessary to expedite the CO_2_RR reaction kinetics. Considering the inactivity of CO_2_ molecules, catalytic strategies like bypassing the formation of CO_2_^•−^ through proton‐assisted multiple‐electron transfer are a promising way to reduce CO_2_ at lower energetic costs.

### 2.2 Possible Reaction Pathways

**Figure 1:**
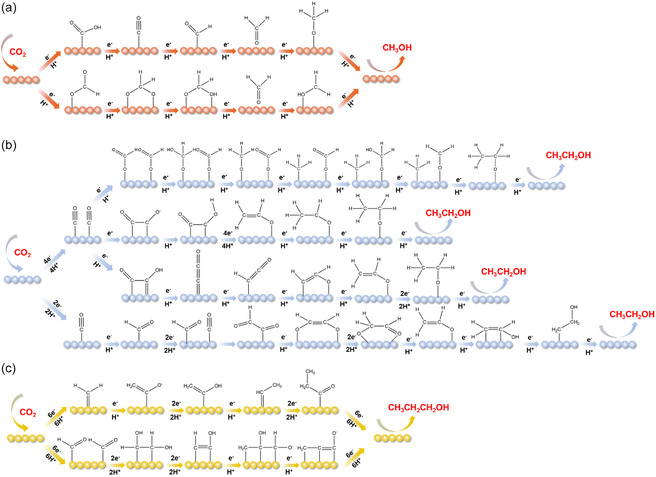
Possible reaction pathways for the CO_2_RR to a) methanol, b) ethanol, and c) propanol.

The electrocatalytic process for CO_2_RR is a complex sequence that typically involves the adsorption of CO_2_, surface diffusion of CO_2_, electron and proton transfer on CO_2_, and the subsequent desorption of the resulting products.^[^ ^52^ ^]^ Possible reaction pathways for the CO_2_RR to alcohols have been summarized in **Figure** **1**.

**Figure 2:**
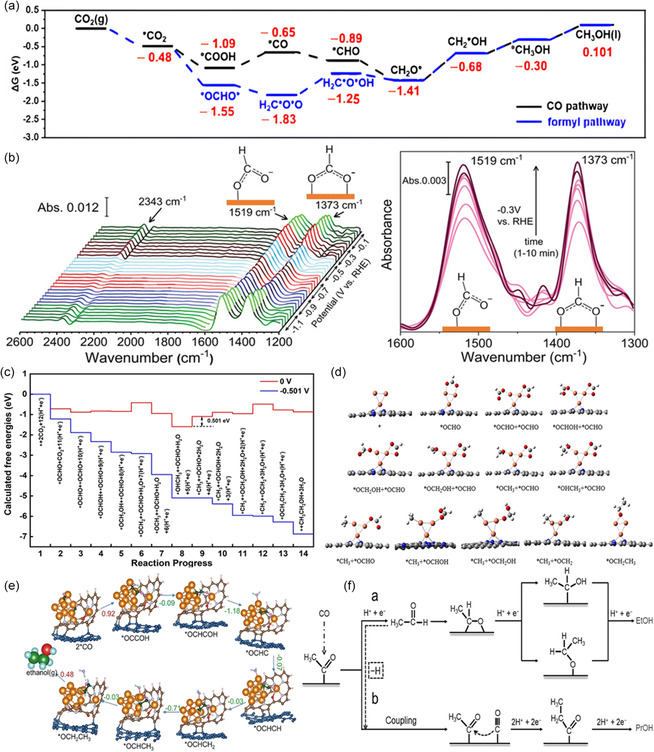
a) Gibbs free‐energy diagrams of CO_2_ to CH_3_OH on the hydroxyl‐modified Mo_2_C NCNT surface at 0 V versus RHE. Reproduced with permission.^[^ ^119^ ^]^ Copyright 2022, The Authors. b) In situ IR spectra were obtained during the CO_2_RR using CuGa_2_ catalyst and IR spectra corresponding to CHO and COO− intermediate formed during CH_3_OH production at a different time during CA at −0.3 V (versus RHE). Reproduced with permission.^[^ ^57^ ^]^ Copyright 2022, Wiley‐VCH. c) Reaction progress of CO_2_ to C_2_H_5_OH on Cu_2_–CuN_3_ at 0 and −0.501 V applied potential. d) Atomic structures of the reaction intermediates along the C_2_H_5_OH pathway. Reproduced with permission.^[^ ^24^ ^]^ Copyright 2022, The Authors. e) Local structures of the active site and intermediate state. Reproduced with permission.^[^ ^59^ ^]^ Copyright 2022, The Authors. f) Pathway for C_2_H_5_OH and C_3_H_7_OH formation. Reproduced with permission.^[^ ^60^ ^]^ Copyright 2020, American Chemical Society.

The reaction pathway of CO_2_RR to CH_3_OH only involves the transfer of 2e^−^ and can be broadly divided into the carboxyl route and formyl route (**Figure** **2**a). In the *COOH pathway of CO_2_RR, the formation of a *CO intermediate occurs by eliminating the hydroxyl group from the *COOH intermediate. Following this, if the adsorption strength of the *CO intermediate is weak, CO dissociates from the catalyst surface as the final product. Conversely, when *CO undergoes hydrogenation, strongly adsorbed *COH or *OCH intermediates are produced. The *OCH conversion process can undergo further hydrogenation to yield *CH_2_OH and *CH_3_OH. These products are eventually desorbed from the catalyst surface, resulting in the formation of CH_3_OH.^[^ ^53^, ^54^, ^55^ ^]^ Li et al.^[^ ^56^ ^]^ reported that a dual‐doping catalyst (Ag, S‐Cu_2_O/Cu) can not only facilitate the formation of *CHO from *CO, but also hinder the HER. This dual action enhances the kinetic efficiency of CO_2_RR towards CH_3_OH. Bagchi and collaborators asserted that the *OCHO pathway is the most likely mechanism for CH_3_OH during CO_2_RR, as supported by in situ IR spectroscopic measurements presented in Figure 2b.^[^ ^57^ ^]^ As the CO_2_RR progresses through the *OCHO pathway, *HCOOH is potentially hydrogenated and dehydroxylated to produce the *OCH_2_ intermediate, subsequently leading to the formation of CH_3_OH through the *OCH in the carboxyl pathway described above. However, *OCHO is predominantly hydrogenated to generate *HCOOH, which may then desorb to produce HCOOH as the final product.

The reaction pathways leading to C_2_H_5_OH are the most complex and controversial compared to other products The prevailing belief is that the pivotal step in reducing CO_2_ to C_2_H_5_OH within the C_2_ pathway involves the formation of a C—C bond through CO_2_ dimerization.^[^ ^58^ ^]^ According to the research conducted by Guo et al.^[^ ^58^ ^]^ the electron‐donating capability of Cu^δ+^ sites is instrumental in efficiently reducing the Gibbs free energy change of the potential‐determining step (Δ*G*_PDS_) of the C—C coupling step, thereby promoting C_2_H_5_OH generation. Regarding the reaction intermediate, Su and co‐workers found that the *OCHO mechanism is more favorable for Cu_2_–CuN_3_ catalysts compared to the *CO mechanisms.^[^ ^24^ ^]^ This preference is attributed to the suppression of CO production concurrent with C_2_H_5_OH formation. In their study, CO_2_ molecules interact with two exposed Cu atoms, forming coadsorbed (*OCHO + *OCHO) species, which undergo C—C bond formation to CH_3_CH_2_OH (Figure 2c,d). CO_2_ can undergo reduction to produce C_2_H_5_OH through the C—C coupling of two CO molecules or CO with intermediates such as *CH and *CH_2_. The *CO intermediate is formed via the carboxyl pathway. Following the coupling of two *CO, the product is hydrogenated and dehydrated to generate a *C_2_O intermediate. A further hydrogenation step produces *C_2_OH or *CHCO. *C_2_OH is progressively hydrogenated to form CH_3_CH_2_OH, while *CHCO is hydrogenated to yield *CH_*x*_CHO (*x* = 1, 2). The latter is then hydrogenated to produce C_2_H_5_OH. Yang et al.^[^ ^59^ ^]^ constructed a hybrid structure of Cu cluster and partially reduced O‐containing hexaphyrin ligand supported by graphene substrate, named R‐Hex‐2Cu‐O/G. Key reaction intermediates for C—C coupling on the catalyst were investigated by highlighting the *CO route of CO_2_RR to C_2_H_5_OH. This process involves two Cu‐adsorbed *CO species before coupling and *OCCOH directly after coupling (Figure 2e). Through DFT calculations, they demonstrated that an additional bond forms between the adsorbed *OCCOH and an adjacent Cu center, enhancing *OCCOH adsorption without affecting the 2*CO adsorption. This disruption of the scaling relation between these carbonaceous intermediates promotes C—C coupling.

Analysis of the production pathways and mechanisms of electrocatalytic CO_2_RR to C_3_H_7_OH is challenging due to the limited literature available. However, based on current research findings, OCCOCO* emerges as the favored *C_3_ intermediate in CO_2_ reduction reactions, a common choice in theoretical calculations. This intermediate not only applies to C_3_H_7_OH but also extends to other products such as CH_3_COCH_3_. Researchers suggested that a minor fraction of CH_3_CHO undergoes dehydrogenative adsorption on the surface, forming a methyl carbonyl species on the OD‐Cu surface (Figure 2f), along with surface hydrogen.^[^ ^60^ ^]^ Adsorbed CO then couples with the methyl carbonyl, followed by hydrogenation steps leading to the formation of C_3_H_7_OH. The study demonstrates that C—C coupling between CO and CH_3_CHO does occur to produce C_3_H_7_OH. However, it is unlikely to be the primary pathway in the CO reduction reaction (CORR). NMR spectra with isotopically labeled reveal that CO attacks the carbonyl carbon of CH_3_CHO in the C—C coupling reaction, with the carbon in CO ultimately being hydrogenated to the hydroxymethyl group (−CH_2_OH) in C_3_H_7_OH.

## 3 Fundamentals of Electrocatalytic CO_2_‐to‐Alcohols Conversion

### 3.1 Configuration of CO_2_RR Devices

#### 3.1.1 H‐Cell

**Figure 3:**
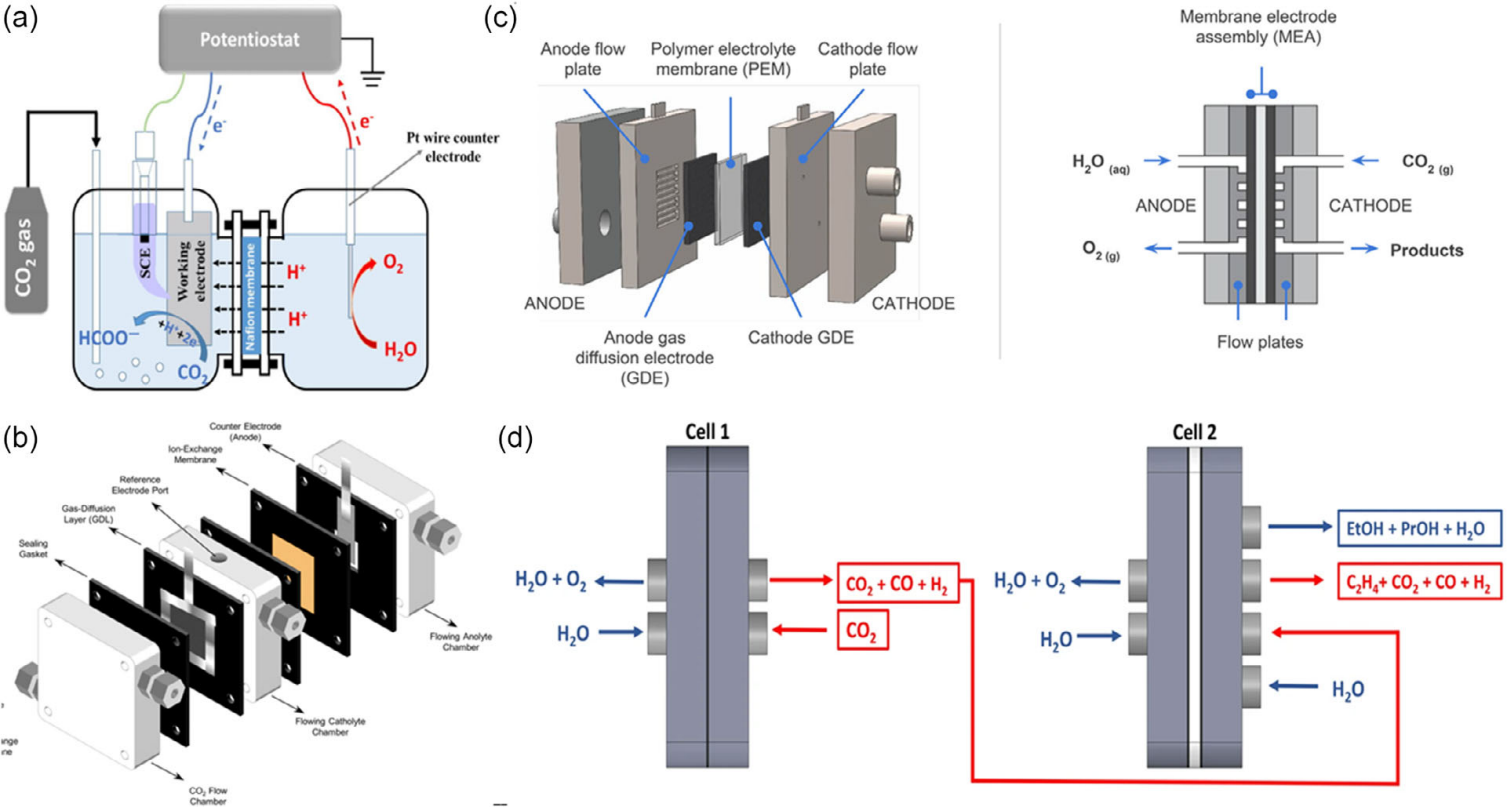
Schematic diagram of different electrolyzers: a) H‐Cell. Reproduced with permission.^[^ ^65^ ^]^ Copyright 2016, Elsevier B.V. b) Flow cell. Reproduced with permission.^[^ ^70^ ^]^ Copyright 2019, American Chemical Society. c) MEA cell. Reproduced with permission.^[^ ^69^ ^]^ Copyright 2019, Elsevier Ltd. d) Tandem electrolyzer system. Reproduced with permission.^[^ ^84^ ^]^ Copyright 2023, The Authors.

Over the past few decades, research on CO_2_RR has focused on the development of effective and selective catalysts using H‐Cell setup, chosen for their straightforward operation, accessibility, and cost‐effectiveness.^[^ ^61^, ^62^, ^63^, ^64^ ^]^ **Figure** **3**a depicts the schematic diagram of the H‐Cell, wherein an ion‐exchange membrane serves as a barrier between the two electrode chambers. This membrane effectively prevents the re‐oxidation of CO_2_RR products on the anode while facilitating the passage of H^+^ to facilitate charge transfer.^[^ ^65^ ^]^ A specific flow rate of CO_2_ gas is consistently purged into the cathodic compartment during electrolysis. The gaseous and liquid products are detected and analyzed through an online gas chromatograph and high‐performance liquid chromatography or NMR, respectively. It is worth noting that achieving optimal detection limits with this instrumentation requires careful consideration of both the duration of the electrolysis process and the cell volume. This is particularly relevant due to the typically low FEs associated with liquid‐phase products.

Despite the promising achievement of FE values exceeding 75% for alcohols, current density values generally remain confined to below 50 mA cm^−2^.^[^ ^47^, ^66^ ^]^ This limitation arises from the inefficient mass transport caused by the low solubility of CO_2_ in water (up to 34 mm at room temperature).^[^ ^67^ ^]^ Furthermore, this challenge is exacerbated by competition with the HER^[^ ^68^ ^]^ and the substantial Ohmic resistance produced by both the ion‐exchange membrane and the dilute electrolyte (usually at 0.1 m). Additionally, elevated voltages within this cell result from the considerable separation (several centimeters) between the anode and cathode, leading to diminished energy efficiency. To address the challenges, flow cell electrolyzers have been proposed.

#### 3.1.2 Flow Cell

In the flow cell configuration, high current densities (>200 mA cm^−2^) can be achieved by a CO_2_ stream as feedstock in the cathode to overcome CO_2_ mass transport limitations.^[^ ^69^ ^]^ The standard configuration of a flow cell typically consists of three chambers, each with specific functions, incorporating a polymer electrolyte membrane that serves to segregate the electrode chambers (Figure 3b).^[^ ^70^ ^]^ To achieve industrially relevant metrics, research has swiftly evolved into gas diffusion electrodes (GDEs). The sustained gas–liquid supply method employed by the flow cell, with the GDE playing a crucial role, offers greater benefits compared to the sporadic operation mode of the H‐cell. The flow cell not only effectively circumvents the CO_2_ mass transfer limitation in the electrolyte solution but also greatly suppresses the HER.^[^ ^71^, ^72^ ^]^ Consequently, enhancing the efficiency of CO_2_RR becomes attainable through the augmentation of CO_2_ mass transfer, which accelerates the apparent kinetics and optimizes product selectivity.^[^ ^73^ ^]^ Moreover, the GDE offers an opportunity for industrial‐scale implementation of CO_2_RR at heightened current densities.^[^ ^74^ ^]^ Nevertheless, obstacles hindering commercialization and industrialization persist, including reported low energy efficiency due to the elevated flow cell resistance, substantial electrolyte cost, and reactor instability. These challenges underscore the ongoing need for research and development efforts to address key technical and economic barriers in realizing the full potential of CO_2_RR technologies.

#### 3.1.3 MEA Cell

In recent years, the MEA has gained prominence as a notable category within the realm of electrochemical cells. As illustrated in Figure 3c, this assembly comprises current collectors for both cathode and anode, flow plates, and a proton exchange membrane, omitting the cathode electrolyte layer. In this particular configuration, the anodic and cathodic electrodes are compressed in conjunction with the ion‐exchange membrane.^[^ ^75^ ^]^ Diverging from H‐Cell and flow cell, wherein the membrane functions as a diaphragm, the membrane in MEA‐Cell serves as a solid electrolyte, creating a zero‐gap structure with a thin‐film thickness at the micrometer level between the cathode and anode. This design serves to effectively minimize the Ohmic resistance, consequently resulting in heightened energy efficiency.^[^ ^76^ ^]^ Moreover, in flow cells, electrocatalysts are conventionally applied onto a gas diffusion layer (GDL) made of carbon paper or carbon cloth. This GDL offers a substantial surface area, facilitating effective contact with CO_2_ molecules and thereby amplifying the maximum limits of CO_2_ reduction rates and current densities.^[^ ^77^ ^]^

Minimizing both mass and electron transfer resistance within the MEA configuration holds the significant potential to augment current densities and energy efficiencies, thereby rendering it more practically viable across diverse applications. Nonetheless, constrained advancements in fabricating high‐performance membranes and challenges in precisely measuring potential due to compact geometry currently restrict the widespread utilization of MEA cell.^[^ ^78^ ^]^

#### 3.1.4 Tandem Electrolysis System

The tandem system refers to the subdivision of the electrocatalytic process into two or more electrolysis reaction cells. This approach allows for the customization of the reaction conditions which can be tailored to the properties of different intermediate products. Consequently, a diverse range of compounds can be synthesized, resulting in higher efficiency and overall productivity. Achieving high FEs in C_2+_ products, as compared to C_1_ products (e.g., CO and HCOOH), at industrially required current densities poses a considerable challenge.^[^ ^79^ ^]^ Investigations have identified CO as the central intermediate in the production of most C_2+_ products.^[^ ^80^, ^81^ ^]^ Through direct electrochemical reduction, CO demonstrates notable selectivity for C_2_H_5_OH at moderate overpotentials and high current densities.^[^ ^82^ ^]^ Guided by these insights, the design and development of a two‐step tandem strategy for C_2+_ products have been undertaken. For example, the initial step involves the conversion of CO_2_ to CO, followed by the subsequent transformation of CO into C_2+_ products in the second step. This tandem approach can be implemented in reaction systems to achieve the desired outcome.^[^ ^83^ ^]^ Möller et al.^[^ ^84^ ^]^ have demonstrated that interconnected tandem electrolyzer cell systems present both kinetic and practical energetic advantages in the direct production of C_2+_ chemicals and fuels from CO_2_ feeds compared to traditional single‐cell systems. Importantly, this process occurs without the necessity for intermediate separation or purification as depicted in Figure 3d. This method results in a 50% augmentation in ethylene production, a 100% escalation in alcohol production, and a significant boost in C_2+_ energy efficiency (by 100%) at current densities reaching up to 700 mA cm^−2^. This improvement is notable when contrasted with the conventional approach of employing a singular CO_2_‐to‐C_2+_ electrolyzer cell system. Similarly, Wu and colleagues devised an efficient two‐step tandem CO_2_RR system, using a 3D single‐atom nickel (3D Ni‐SAG) for CO_2_‐to‐CO conversion and multihollow Cu_2_O for the subsequent CO_2_‐to‐C_3_H_7_OH.^[^ ^85^ ^]^

Tandem electrolyzer configurations are anticipated to enhance the kinetics of sluggish catalytic reactions by furnishing adaptable reaction environments within distinct cells. This tandem methodology harbors the capability to advance the evolution of modular industrial apparatus for CO_2_RR. Such modules can be seamlessly integrated to facilitate continuous and proficient industrial manufacturing. The adoption of this tandem strategy not only enhances conversion rates but also improves the purity of the resultant products, thereby emphasizing the auspicious and pragmatic applications inherent in this innovation approach.

### 3.2 Electrolytes for CO_2_RR to Alcohols

**Figure 4:**
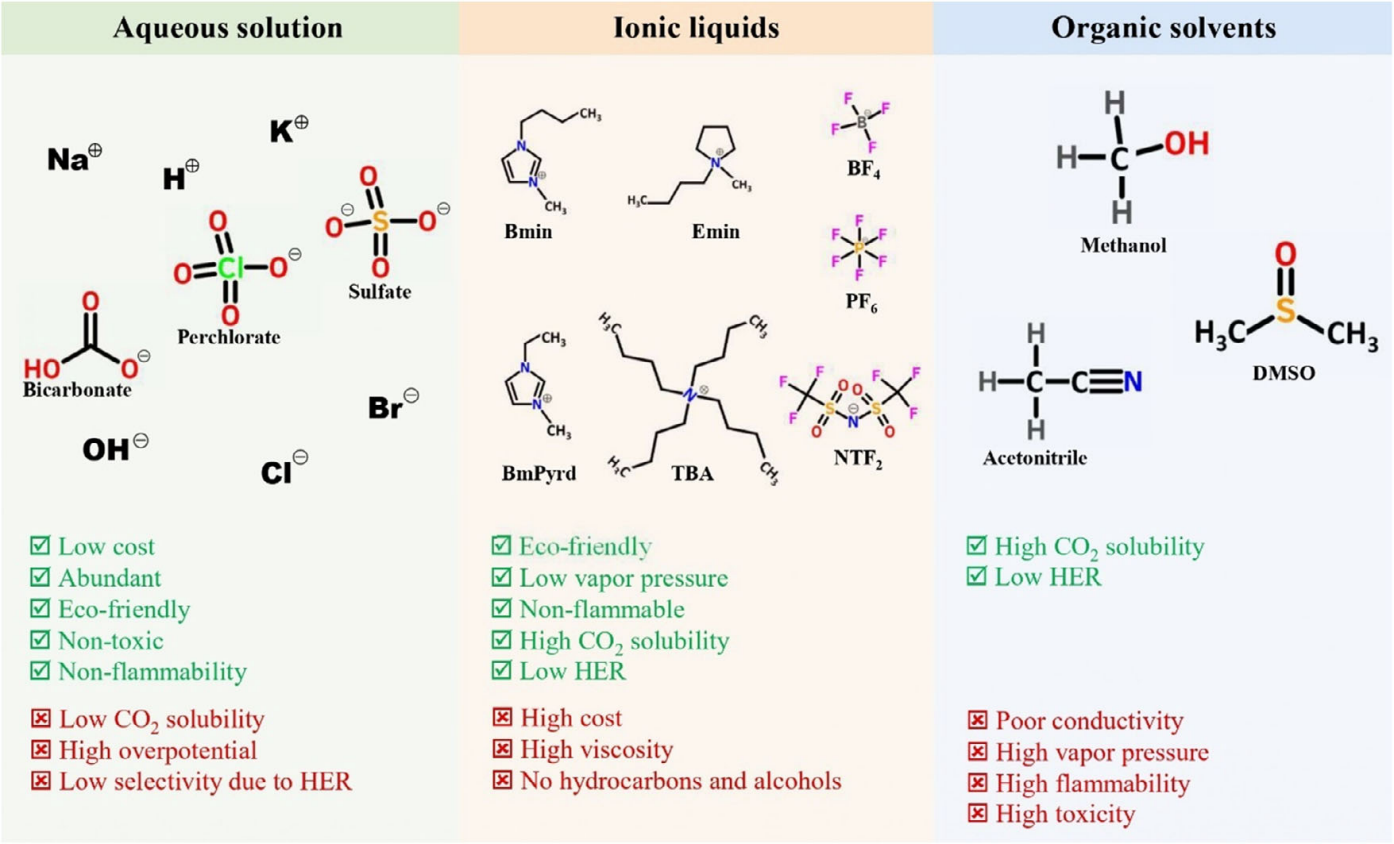
Comparison of electrolyte families used for CO_2_RR.

The significance of electrolytes in electrocatalytic systems for CO_2_ reduction is noteworthy. Acting as a conduit for transferring both electrons and protons in CO_2_RR, the nature and concentration of electrolytes play a crucial role in influencing catalyst activity and selectivity. Additionally, specific cations or anions in the electrolyte can either stabilize reaction intermediates or hinder their formation, potentially playing a direct role in determining the preferred reaction pathways.^[^ ^86^ ^]^ This section outlines the application and effects of electrolytes (including aqueous solutions, ILs, and organic electrolytes) for the CO_2_RR (**Figure** **4**).

#### 3.2.1 Aqueous Solution Electrolytes

Most investigations on electrochemical CO_2_ reduction use aqueous electrolytes. Aqueous solutions stand out as the preferred choice for electrochemical gas sensors due to their affordability, variety, user‐friendliness, and general performance. Nevertheless, the limited solubility of CO_2_ in water results in reduced concentrations of CO_2_ in saturated aqueous electrolytes.^[^ ^87^ ^]^ This limitation primarily hampers the reaction kinetics due to the formation of CO_2_^•−^ radical.^[^ ^88^ ^]^ Consequently, substantial overpotentials are typically necessary to drive the reaction. A more troublesome factor is the ubiquity of HER in aqueous environments. Serving as a competitive reaction, the HER has the potential to reduce the selectivity of the desired product and decrease the FE of CO_2_RR.^[^ ^89^ ^]^

**Figure 5:**
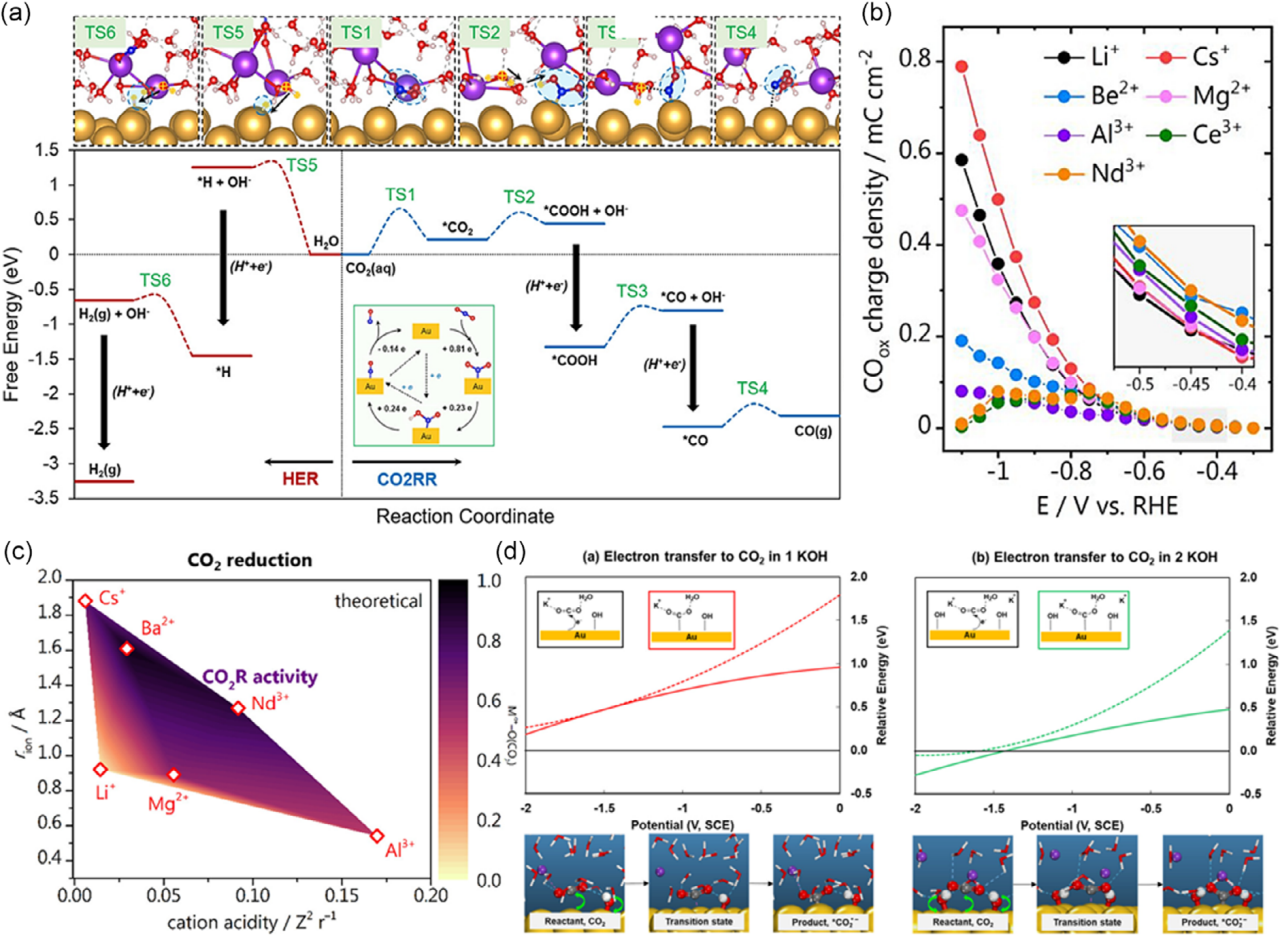
a) Complete free‐energy landscape of CO_2_RR and competitive HER at Au–water interfaces with two K cations. Reproduced with permission.^[^ ^171^ ^]^ Copyright 2023, American Chemical Society. b) Amount of CO produced probed via consecutive cathodic/anodic voltammetry at pH 3 in 0.1 m Li_2_SO_4_ + 1 mm M^n+^ electrolytes with M^n+^ = Li^+^, Cs^+^, Be^2+^, Mg^2+^, Al^3+^, Nd^3+^, Ce^3+^. c) CO_2_ reduction activity predicted assuming average cation–CO_2_ coordination (N_M_^n+^_–O(CO2)_) as a potential descriptor versus ionic radius and cation acidity. Reproduced with permission.^[^ ^93^ ^]^ Copyright 2021, The Authors. d) Structures and energetics for the free CO_2_ reactant, transition state (TS), and product *CO_2_^•−^ during adsorption in 1 KOH and 2 KOH solutions on Au(111). Reproduced with permission.^[^ ^97^ ^]^ Copyright 2023, American Chemical Society.

Electrochemical reduction of CO_2_ commonly utilizes aqueous electrolytes that are either weakly acidic or alkaline. These electrolytes are typically CO_2_ saturated and include inorganic salts featuring anions such as HCO_3_^−^, SO_4_^2−^, or Cl^−^, along with alkali metal cations such as Na^+^ and K^+^. Research indicates that high concentrations of cations, specifically K^+^, play a crucial role in restraining hydrogen precipitation reactions and triggering the activation of CO_2_ within acidic environments.^[^ ^90^, ^91^ ^]^ As shown in **Figure** **5**a, achieving CO_2_ reduction in the presence of K^+^ is facilitated at the inner‐sphere interface, where the activation of CO_2_ occurs with a minimal free energy barrier of only 0.66 eV. This observed phenomenon may be attributed to the establishment of an electric double‐layer field spanning the Helmholtz layer. This field is instrumental in stabilizing intermediates (such as *CO_2_) through interactions with the adsorbate dipole field. The extent of alkali metal cation (M^+^) accumulation at the interface can dynamically influence and modulate this interaction.^[^ ^92^ ^]^ Furthermore, cationic entities can modify the potential at the outer Helmholtz plane (OHP) and can impact the coverage of hydrogen on the electrode by transporting water molecules from their solvation shell to the electrode. The presence of interfacial cations hinders the competitive HER through an induced kinetic blocking effect. In this scenario, the Volmer step, which determines the rate‐limiting stage, encounters a significantly elevated energy barrier of 1.27 eV.

Utilizing explicit electric fields in molecular dynamics simulations reveals a phenomenon wherein nonacidic cations experience reduced repulsion at the interface. As a result, these cations tend to accumulate more prominently at the OHP (interface), thereby initiating localized promoting effects.^[^ ^93^ ^]^ The data in Figure 5b indicates a clear trend wherein higher activity for CO_2_RR‐to‐CO is observed in electrolytes containing trivalent cations, followed by divalent and monovalent cations (at −1 V versus RHE). Theoretical considerations highlight Cs^+^ and Ba^2+^ as yielding the best performance at high overpotentials, while Nd^3+^ appears to be optimal for the low‐overpotential region. This evaluation relies on the mean coordination number between cations and CO_2_, serving as the exclusive indicator of CO_2_ reduction activity when the competing water reduction reaction is not present, as depicted in Figure 5c. In the absence of a metal cation, gold, copper, or silver electrodes were observed to be incapable of forming any CO according to research findings.^[^ ^94^ ^]^ On the contrary, Bhargava et al.^[^ ^95^ ^]^ recently documented that the electrochemical reduction of CO_2_ to CO on silver GDEs faces hindrance from multivalent cations. This hindrance is linked to the formation of deposits that block active sites on silver. Simultaneously, cations have the potential to influence the concentrations of charged species, such as anion radical intermediates, in proximity to the electrode. This influence can consequently impact product selectivity and current density.

Varieties of anionic species, including Cl^−^, ClO_4_^−^, SO_4_^2−^, HCO_3_^−^, and H_2_PO_4_^−^, exhibit diverse buffer capacities that influence the localized pH near the electrode. This alteration in pH has consequential effects on the catalyst's morphology, thereby impacting the adsorption energy and binding strength of intermediates to the electrode surface. Additionally, this leads to the obstruction of active sites which are crucial for the adsorption of both reactants and intermediates.^[^ ^96^ ^]^ The OH^−^ ions in the solution can adsorb onto the Au cathode, reaching potentials as low as approximately −3 V (versus saturated calomel electrode). This adsorption allows the OH– ions to transfer electrons to the Au cathode and enter the antibonding 2π* orbitals of CO_2_. Consequently, this process facilitates the crucial steps of adsorption and electron transfer to CO_2_, leading to the formation of the adsorbed *CO_2_^•−^ radical anion (Figure 5d).^[^ ^97^ ^]^

#### 3.2.2 Ionic Liquids (ILs) as Electrolytes

**Figure 6:**
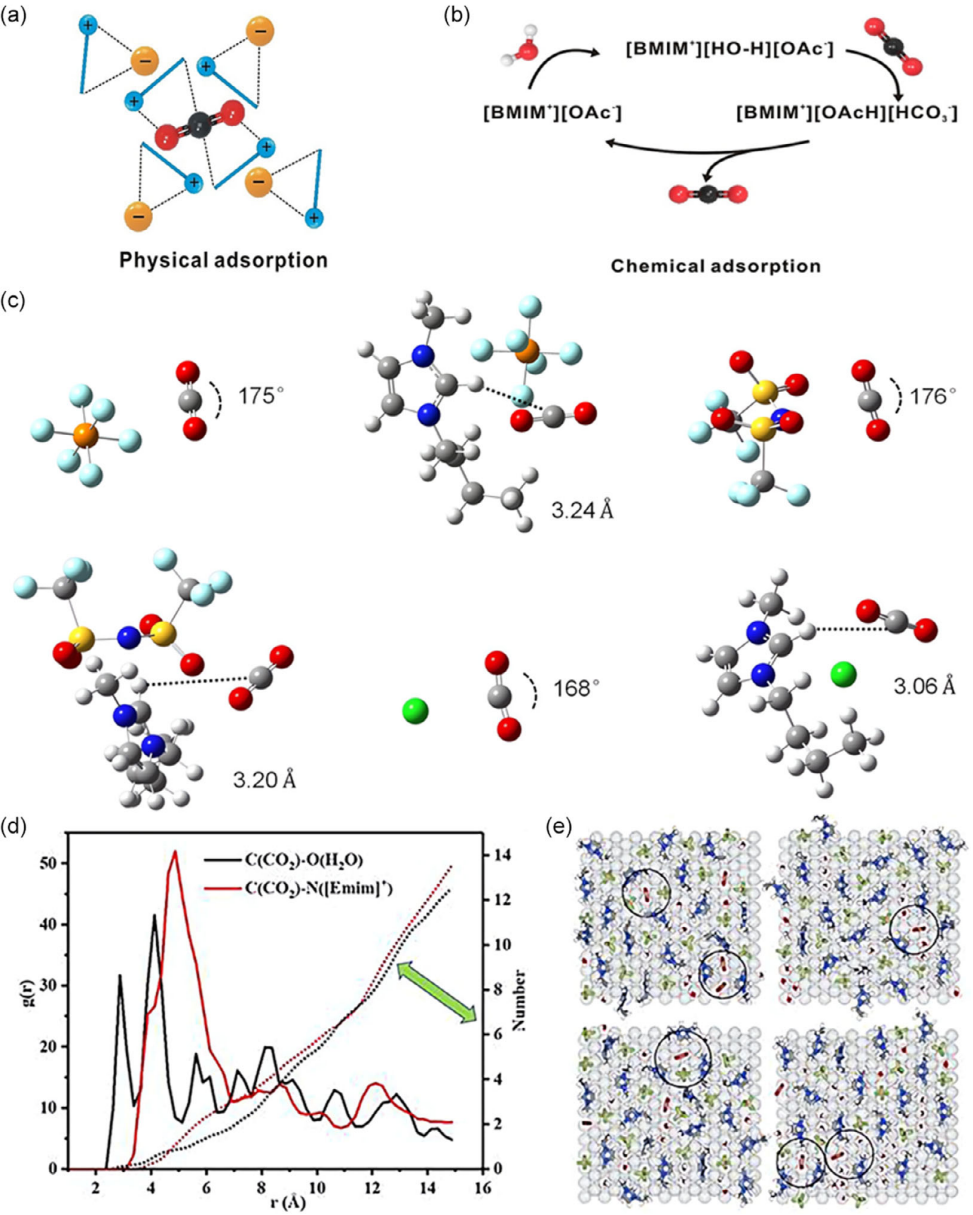
a) Physical adsorption of CO_2_, mainly in ILs with nonbasic nucleophilic anions. b) Chemical adsorption of CO_2_, mainly in ILs with acid protons for easy deprotonation and basic anions. Reproduced with permission.^[^ ^103^ ^]^ Copyright 2021, Wiley‐VCH. c) Optimized geometries of the [PF_6_^−^]…CO_2_, [BmimPF_6_]…CO_2_, [NTF_2_^−^]…CO_2_, [BmimNTF_2_]…CO_2_, [Cl‐]…CO_2_ and [BmimCl]…CO_2_. Reproduced with permission.^[^ ^100^ ^]^ Copyright 2022, Elsevier B.V. d) Radial distribution function between C atoms of CO_2_ molecules, N atoms of cations, and O atoms of H_2_O, and a number of [Emim]^+^ cations and H_2_O around one CO_2_ molecule. e) Typical snapshots of the first‐layer molecules near the Ag(110) surface. Reproduced with permission.^[^ ^172^ ^]^ Copyright 2022, Elsevier B.V.

ILs, commonly referred to as molten salts at room temperature, are typically composed of organic cations and anions, forming salt compounds that remain in a liquid state under normal environmental conditions. The process of CO_2_ dissolution in ILs encompasses two primary mechanisms: physical adsorption (**Figure** **6**a) and chemical adsorption (Figure 6b). Physical adsorption predominantly takes place in ILs featuring nonbasic nucleophilic anions such as bis(trifluoromethyl sulfonyl)amide. In such cases, CO_2_ is confined within cavities near alkyl groups and aromatic protons of the ILs, establishing a weak interaction that does not disrupt the hydrogen bonds between cations and anions. This interaction only leads to minimal changes to the structure of the IL. On the other hand, chemical absorption of CO_2_ primarily occurs through carboxylation, wherein CO_2_ undergoes conversion into bicarbonate. This form of CO_2_ sorption is observed in media rich in protons with acidic characteristics, facilitating deprotonation and featuring basic anions such as acetate and imidazolium.

The utilization of ILs, particularly those incorporating imidazolium cations, offers advantages in multiple aspects: 1) reducing the overpotential for CO_2_ reduction, possibly through complexation to lower the energy of the CO_2_^•−^ intermediate; 2) suppressing the HER; 3) enhancing the selectivity for the formation of the desired target product, and 4) increasing the solubility of CO_2_.^[^ ^98^, ^99^ ^]^ Nickel foam electrodes exhibit enhanced charge transportation when immersed in a solution of 0.5 m [Bmim]Cl/MeCN.^[^ ^100^ ^]^ Quantum chemical calculations indicate a robust interaction between the chloride anion of [Bmim]Cl and CO_2_, resulting in the bending and activation of the CO_2_ molecule, as illustrated in Figure 6c. The structural analysis reveals that most CO_2_ molecules readily bond with the cations, causing the O atoms to realign toward the H atoms on the imidazolium ring. Notably, this realignment is more pronounced around H (C_4/5_) rather than H (C_2_) and H (H_2_O). This distribution of structure suggests that the ILs establish synergistic catalytic mechanisms involving multiple ions within a mesoscale microenvironment, as illustrated in Figure 6d,e.

In recent investigations, Li and co‐authors successfully achieved a groundbreaking milestone in their research, surpassing a current density of 100 mA cm^−2^ for the first time.^[^ ^56^ ^]^ This accomplishment was realized through the development of the Ag, S–Cu_2_O/Cu electrocatalyst, coupled with [Bmim][BF_4_]/H_2_O as the electrolyte, yielding an impressive value of 122.7 mA cm^−2^. When ILs were incorporated into the cathode catalyst, the cell operating current experienced a twofold or greater increase, leading to a substantial improvement in FE.^[^ ^101^ ^]^ However, there are crucial factors that may hinder their industrial‐scale use, including their high cost and viscosity.^[^ ^98^ ^]^ The electrolyte's conductivity improves as the concentration of ionic species rises due to an increase in the concentration of ILs. In contrast, the addition of ILs also raises the viscosity of the electrolyte, leading to a decrease in conductivity.^[^ ^102^ ^]^

#### 3.2.3 Organic Solvents

In contrast to the enthusiasm surrounding aqueous and IL electrolytes, scholars have exhibited comparatively less interest in organic solvents. However, recent investigations have delved into the utilization of electrolytes containing organic solvents characterized by higher boiling points and dissolved inorganic salts. This approach addresses the challenge of electrolyte dryness by leveraging their robust chemical stability, broad redox potential window, elevated boiling points, and low volatility of these solvents. Furthermore, the use of organic solvents in the CO_2_RR process leads to an extended cathodic potential window due to the inhibition of the HER, which is a result of the absence of protons compared to aqueous environments. Consequently, electrocatalytic reactions can be conducted over a wider potential range.^[^ ^103^, ^104^, ^105^ ^]^ Han and co‐workers synthesized Mo–Bi chalcogenides to catalyze the electrochemical reduction of CO to CH_3_OH, and an FE of 71.2% for CH_3_OH was obtained at a current density of 12.1 mA cm^−2^.^[^ ^102^ ^]^ Unfortunately, the practical applicability of this achievement is restricted due to the reliance on an organic solvent as the electrolyte, with the reaction specifically conducted in CH_3_CN. Besides, the choice of organic electrolyte is of great importance as it exerts a significant influence on the reaction mechanism, ultimately affecting the selectivity and product distribution.^[^ ^106^ ^]^ However, a substantial gap remains in research regarding the influence of various organic electrolytes for CO_2_RR to alcohols.

### 3.3 Electrocatalysts for CO_2_RR to Alcohols

Currently, diverse catalysts have been developed to enhance the activity and/or selectivity of the CO_2_RR. Encouragingly, many of these catalysts exhibit high efficacy in producing alcohols, thus significantly advancing the development of sustainable energy solutions. This section examines the latest advancements in prominent electrocatalysts for the electrochemical CO_2_ conversion into methanol, ethanol, and propanol respectively.

#### 3.3.1 Catalysts for Methanol Generation

Generally, the reported FE_MeOH_ for CO_2_‐to‐CH_3_OH electrocatalytic conversion is relatively low due to the poor selectivity of the catalysts.^[^ ^107^, ^108^ ^]^ A list of representative efficient catalysts proposed is presented in **Table** **1**. These catalysts encompass diverse structural configurations, including SACs, heterostructure, metal alloy catalysts, and others.

**Table 1:** Summary of representative electrocatalysts used for CO_2_ reduction to methanol since 2020.

Category	Electrocatalysts^[ref.]^	Electrolyte	Potential (V versus RHE)	FE [%]	*J* [mA cm^−2^]
SACs	Ti_3_(Al_1−*x*_Cu_*x*_)C_2_^[^ ^115^ ^]^	0.1 m KHCO_3_	−1.4	59.1	~10
	B‐CoPc‐400^[^ ^116^ ^]^ (CORR)	0.5 m KOH	−0.8	50	35
	CoPc/MWCNT^[^ ^7^ ^]^	0.25 m K_2_HPO_4_	−2.9	65	30
	Ni‐2D–O–SA–CNT^[^ ^173^ ^]^	0.1 m KHCO_3_	−0.9	27	0.94
	Cu_3_(HHTQa))_2_^[^ ^174^ ^]^	0.1 m KHCO_3_	−0.4	53.6	–
	Sn_1_/V_o_–CuO–xb) ^[^ ^175^ ^]^	([Bmim]BF_4_): H_2_O = 1: 3	−2.0 (versus Ag/Ag^+^)	88.6	67
	CuSAs/TCNFs^[^ ^53^ ^]^	0.1 m KHCO_3_	−0.9	44	93
Heterostructure	CoO/CN/Ni^[^ ^176^ ^]^	0.5 m KHCO_3_	−0.7	70.7	10.6
	Mo_2_C NCNT^[^ ^119^ ^]^	0.1 m KHCO_3_	−1.1	80.4	>4
	RuOMc)‐CNTs^[^ ^177^ ^]^	0.5 m NaHCO_3_	−1.35	65	–
	Cu_2_NCN^[^ ^22^ ^]^	0.5 m KHCO_3_	–	70	92.3
	Cu@Cu_2_O‐400 °C^[^ ^120^ ^]^	0.5 m KHCO_3_	−0.7	45	–
	Pd_1.80%_/MnO_2_^[^ ^121^ ^]^	1 m KOH	−0.6	80.9	243.5
	Pt/PANI@ZnO^[^ ^122^ ^]^	3 m KCl	−1.09	57	15
	Ag, S‐Cu_2_O/Cu^[^ ^56^ ^]^	([Bmim]BF_4_): H_2_O = 1:3	−1.18	67.4	122.7
	ZnO–Cu−C_60_^[^ ^178^ ^]^	BmimBF_4_/H_2_O	−0.63	78.3	20.3
Molecular catalysts	Co Corrole^[^ ^179^ ^]^	0.1 m NaClO_4_	−0.64	45	–
	CoPc/CNT^[^ ^180^ ^]^	0.1 m KHCO_3_	−0.94	>40	30
	Cu/Bi‐MOF^[^ ^181^ ^]^	0.5 m KHCO_3_	−0.21	18.2	10
Intermetallic alloys and metal oxides	CuGa_2_^[^ ^57^ ^]^	0.5 m KHCO_3_	−0.3	77.26	21.4
	Pt_*x*_Zn/C (1 < *x* < 3)^[^ ^124^ ^]^	0.1 m NaHCO_3_	−0.9	81.4	–
	CuO NW^[^ ^182^ ^]^	1 m KOH	−1.4 (versus Ag/Ag^+^)	66.4	≈13

a) HHTQ: hexahydroxytricyclo‐quinazoline.

b) x: the H‐plasma treatment time in seconds.

c) OM: organometallic.

##### Single‐Atom Catalysts (SACs)

**Figure 7:**
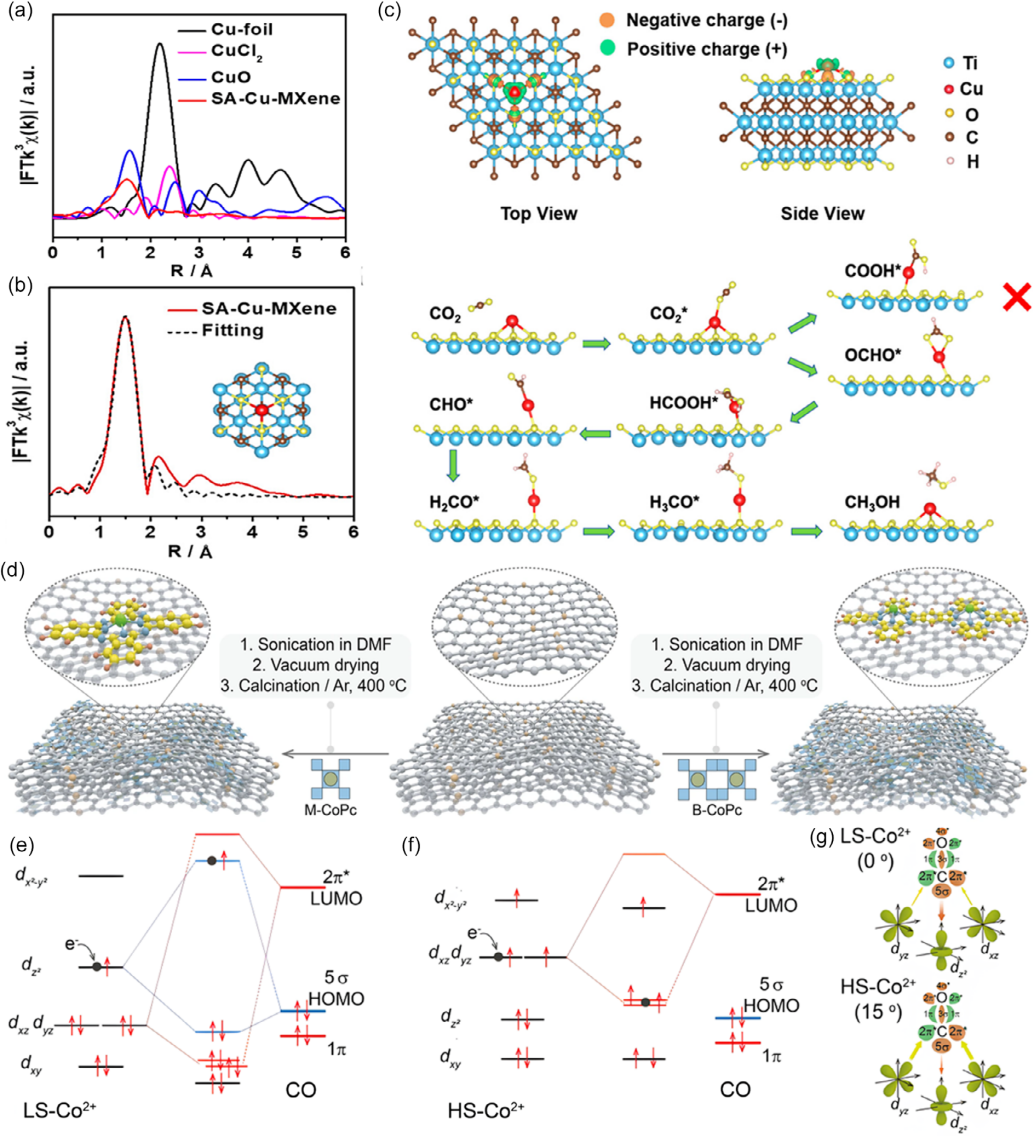
a) *k* ^3^‐weighted Fourier transform of the extended X‐ray absorption fine structure (EXAFS) spectra of SA‐Cu‐MXene compared with Cu foil, CuO, and CuCl_2_ references. b) EXAFS curves between the experimental data and the fit of SA‐Cu‐MXene. c) DFT calculations of charge density difference of SA‐Cu‐MXene and a reaction pathway for the functionalization of CO_2_ to CH_3_OH on isolated Cu of SA‐Cu‐MXene. Reproduced with permission.^[^ ^115^ ^]^ Copyright 2021, American Chemical Society. d) Schematic of the synthesis process for M‐CoPc‐RT/400 and B‐CoPc‐RT/400. Interactions between CO molecular frontier orbitals (5*σ* and 2π*) and the 3*d* orbital of e) LS‐Co^2+^ and f) HS‐Co^2+^ site. g) Schematic of σ and π‐donation bonds between CO and 3*d* orbital of low‐spin Co^2+^ (LS‐Co^2+^) and high‐spin Co^2+^ (HS‐Co^2+^). Reproduced with permission.^[^ ^116^ ^]^ Copyright 2023, The Authors.

SACs possess distinct, isolated active centers, serving as an exemplary model system that bridges the gap between homogeneous and heterogeneous catalysis.^[^ ^109^, ^110^ ^]^ Thus, by featuring individual metal atoms scattered across solid substrates, SACs leverage the strengths of both homogeneous and heterogeneous catalysts. The near‐maximum atom utilization efficiency and the unsaturated coordination environment not only mitigate the elevated synthesis costs arising from excessive metal use but also boost the overall catalytic performance of these catalysts, often exhibiting higher activity and selectivity compared to mixed metal systems.^[^ ^111^ ^]^ The valence electrons within the d‐state of a metal atom in SACs exhibit proximity to the Fermi level, facilitating rapid electron transfer and enhancing the adsorption of CO_2_ intermediates, which addresses the challenges posed by high activation barriers and sluggish kinetics in CO_2_RR.^[^ ^112^, ^113^, ^114^ ^]^ The top‐down approach involves confining individual active atoms within a specific region of the layered structures. Subsequently, these structures are exfoliated to yield ultrathin 2D layers, maximizing the exposure of active atoms. This method has emerged as an ideal strategy to produce SACs with highly exposed catalytically active sites and exceptional activities across a range of reactions. Zhao et al.^[^ ^115^ ^]^ synthesized MXene layers with immobilized single‐atom copper by selectively etching atomic‐level hybrid A layers (Al and Cu) from quaternary MAX phases (Ti_3_(Al_1−*x*_Cu_*x*_)C_2_). The resulting single‐atom Cu catalyst demonstrates a high FE value of 59.1% in the production of CH_3_OH and exhibits robust electrocatalytic stability. Through XAS analysis (**Figure** **7**a,b) and DFT calculations (Figure 7c), it was revealed that the single‐atom Cu possesses an unsaturated electronic structure (Cu^δ+^, 0 < *δ* < 2). This unique electronic configuration contributes to a low‐energy barrier for the rate‐determine step (conversion of HCOOH* to absorbed CHO* intermediate), resulting in the efficient electrocatalytic reduction of CO_2_ to CH_3_OH. Ren et al.^[^ ^7^ ^]^ demonstrated that in tuning the ligand structure and optimizing the interaction between cobalt phthalocyanine (CoPc) and supports in a model, Co‐SAC results in the generation of more reduction products such as CH_3_OH. Theoretical calculations evidenced the low‐energy barrier required for the formation of the CO_2_^•−^ intermediate which is a key aspect in driving the electrochemical reduction of CO to methanol. Based on that, Ding and co‐workers^[^ ^116^ ^]^ constructed two model catalysts by anchoring cobalt phthalocyanine and binuclear cobalt phthalocyanine (M‐CoPc and B‐CoPc) on nitrogen‐doped carbon support (Figure 7d). Having well‐defined coordination structures, these systems offer an ideal model to reveal the catalyst structure–performance relationship. The CH_3_OH partial current density achieved ≈35 mA cm^−2^ at −0.8 V (versus RHE) with FE of 50%. The improved CO reduction performance can be attributed to the electron rearrangement of the Co 3*d* orbitals induced by the change of molecular conformation (Figure 7e–g).

##### Heterostructure Catalysts

Heterostructure catalysts designed for CO_2_ electrochemical reduction have gained significant recognition and are increasingly becoming a focal point in this field. Heterostructures are materials composed of multiple phases, featuring interfaces between different components.^[^ ^117^ ^]^ The incorporation of multiple constituents establishes a synergistic effect, enhancing the catalyst's performance and demonstrating considerable potential in electrocatalytic applications.^[^ ^118^ ^]^ In the context of CO_2_RR, heterostructure electrocatalysts offer a pathway to achieving superior performance, high selectivity, and enhanced stability.

**Figure 8:**
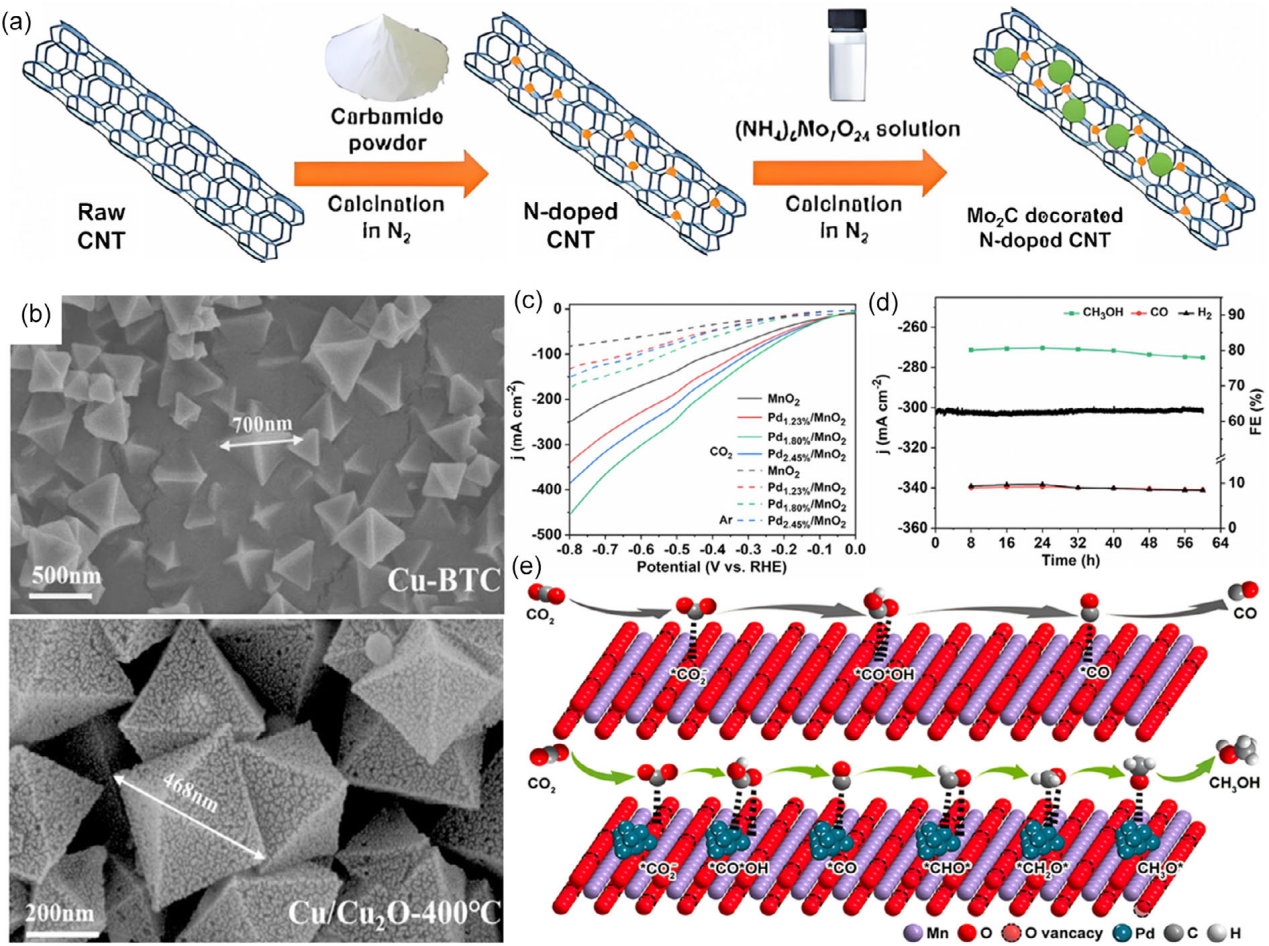
a) Schematic illustration for the preparation of Mo_2_C NCNT. Reproduced with permission.^[^ ^119^ ^]^ Copyright 2022, The Authors. b) Scanning electron microscopy (SEM) images of Cu‐BTC and Cu@Cu_2_O electrocatalysts derived from Cu‐BTC pyrolysis at 400 °C. Reproduced with permission.^[^ ^120^ ^]^ Copyright 2021, Elsevier B.V. c) Linear sweep voltammetry curves in 1.0 m KOH electrolyte with CO_2_ or Ar gas supplied to the gas chamber. d) Chronopotentiometry test and corresponding FE of CH_3_OH, CO, and H_2_ on Pd_1.80%_/MnO_2_ at −0.6 V for 60 h. e) Schematic illustration of the catalytic mechanism toward CO_2_RR to CH_3_OH over Pd/MnO_2_. Reproduced with permission.^[^ ^121^ ^]^ Copyright 2023, American Chemical Society.

The utilization of conductive carbon‐based materials as a support for the integration of metal nanoparticles (NPs) has been extensively explored, which is commonly employed to synthesize heterostructured electrocatalysts with exceptional intrinsic properties. A novel strategy to control the adsorption energy of CO_2_ reduction intermediates on Mo_2_C/‐N‐doped carbon nanotube (N‐CNT) through metal–carbon hybridization has been proposed, as shown in **Figure** **8**a.^[^ ^119^ ^]^ The electronic interaction between Mo_2_C and N‐CNT facilitates the formation and conversion of oxygen‐bound intermediates by boosting the adsorption energy of oxygen atoms, which enhance the CO_2_‐to‐CH_3_OH conversion. Of note, carbon materials doped with electron‐donating heteroatom elements (e.g., N) have emerged as attractive substrates due to their flexible electronic properties. These materials can effectively interact with metals, promoting electron‐rich localization in catalytic active sites and inducing alterations to the electronic structure. Consequently, this optimization enhances the binding and stabilization of CO_2_ or associated intermediates along the reduction pathways. The improved ease of activation and reduction led to remarkable specific activity and selectivity, achieving a high FE of 80.4% for CH_3_OH at −1.1 V versus RHE.^[^ ^119^ ^]^ In addition, a dual doping strategy is considered to construct efficient CO_2_‐to‐CH_3_OH electrocatalysts.^[^ ^56^ ^]^ Their study suggests that the anion S can effectively adjust the electronic structure and morphology of the catalysts in favor of the methanol pathway, whereas the cation Ag suppresses the HER. Besides carbon/metal composites, integrating different electroactive metal components is effective for constructing efficient heterostructured catalysts. Yang et al.^[^ ^120^ ^]^ developed a novel MOF‐derived Cu@Cu_2_O heterogeneous electrocatalyst (Figure 8b) with moderate intermediate adsorption and proposed it for highly selective reduction of CO_2_ to CH_3_OH. This system exhibited a peak FE_MeOH_ of 45% at −0.7 V which was attributed to the synergistic effect between Cu^0^ and Cu^+^ active sites. Unfortunately, most catalyst produces more than one liquid product other than CH_3_OH, increasing the cost of downstream separation. In response to this challenge, Zhu and co‐workers devoted to designing a composite catalyst for CO_2_RR with high selectivity at a large current density, leading to the exclusive formation of CH_3_OH as a single C_1_ liquid product.^[^ ^121^ ^]^ The integrated MnO_2_ nanosheets with Pd NPs (Pd_1.80%_/MnO_2_) exhibited the highest catalytic activity and exceptional stability for CO_2_RR to CH_3_OH (Figure 8c,d), which is attributed to the modification of the electronic structure of MnO_2_ nanosheets by Pd NPs and the generated oxygen vacancies rich on MnO_2_ lattices (Figure 8e).

Peculiarly, Khalili et al.^[^ ^122^ ^]^ tried to produce value‐added products and decrease energy consumption by combining the electroreduction of CO_2_ with wastewater electro‐oxidation processes. The FE for CH_3_OH production and total cathodic energy efficiency were 57% and 34%, respectively, for the 4 h durability test at the cathodic potential of −1.09 V versus Ag/AgCl. Simultaneously for the β‐PbO_2_ electrode, the chemical oxygen demand removal reached up to 93% with a specific energy consumption of 4.6 kWh m^−3^. The paired process provides new ideas for improving the technical and economic evaluation of CO_2_RR.

##### Intermetallic Alloys Catalysts

**Figure 9:**
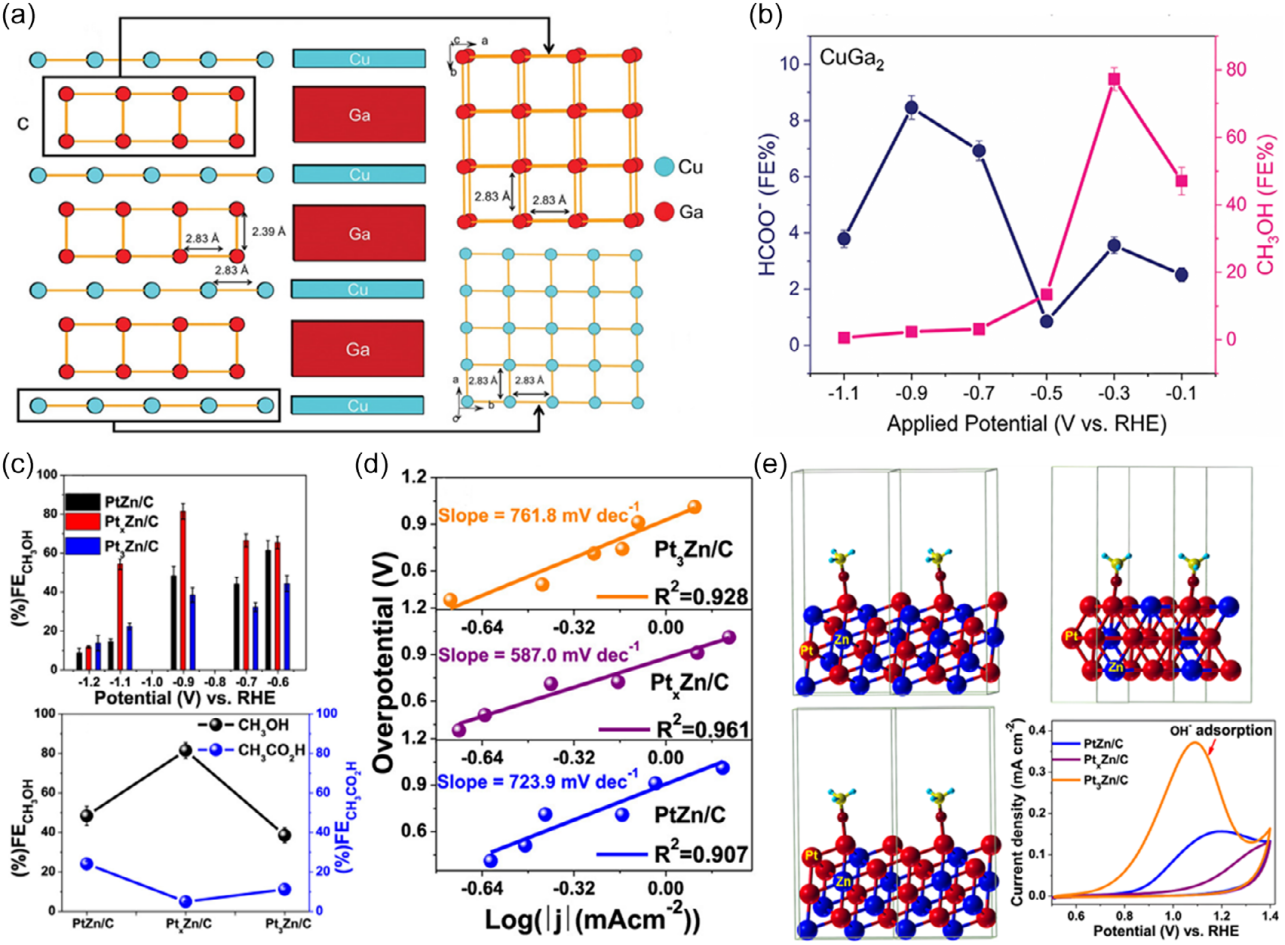
a) Crystal structure of Cu–Ga‐based intermetallic. b) FE for each CO_2_‐reduced liquid product CH_3_OH and formate as a function of potential during CO_2_RR on CuGa_2_. Reproduced with permission.^[^ ^57^ ^]^ Copyright 2022, Wiley‐VCH. c) FE of CH_3_OH in CO_2_RR over the intermetallic nanoalloys as a function of potentials and comparison of FEs for CH_3_OH and CH_3_CO_2_H at −0.90 V over the three intermetallic nanoalloys. d) Tafel plots over the intermetallic nanoalloys. e) (111) plane of PtZn, Pt_3_Zn, and Pt_*x*_Zn with adsorbed *–OCH_3_, and CV traces for hydroxide adsorption in 0.1 m NaOH solution over the intermetallic nanoalloys. Reproduced with permission.^[^ ^124^ ^]^ Copyright 2020, American Chemical Society.

In addition to SACs and heterostructure catalysts, intermetallic alloys stand out in electrocatalysis due to their distinct stoichiometry, precise atomic arrangement, and meticulously controlled crystal structure.^[^ ^123^ ^]^ Consequently, they have become a compelling area of research at the forefront of CO_2_RR. Unlike conventional alloys characterized by random atomic ordering, intermetallic alloys with unique geometric and electronic structures showcase significantly improved catalytic performance. However, overdependence on expensive precious metal catalysts makes efficiently converting CO_2_ into CH_3_OH with extremely low energy input an environmentally feasible process. A Ga‐based material for CH_3_OH selectivity upon CO_2_RR at ultralow applied potential has been reported for the first time.^[^ ^57^ ^]^ A 2D CuGa_2_ crystal structure (**Figure** **9**a) was designed by high‐temperature solid‐state reaction, which can selectively convert CO_2_ to CH_3_OH with a remarkable FE of 77.26% and a current density of 21.4 mA cm^−2^ at an extremely low potential of −0.3 V versus RHE. This particular structural arrangement characterized by a more exposed surface offers advantages for enhancing the accessibility of additional metal atoms to the surface. Their investigation revealed that, at higher potentials, the oxide layer undergoes reduction to metal, resulting in a shift in the mechanism that compromises the catalyst's efficiency and consequently reduces the FE of CH_3_OH (Figure 9b).

Alloy structures and the interface between different metals have obvious effects on CO_2_RR. Payra et al.^[^ ^124^ ^]^ synthesized PtZn/C, Pt_3_Zn/C, and Pt_*x*_Zn/C (1 < *x* < 3) from metal–organic framework materials to demonstrate the CO_2_RR performance for CH_3_OH production and elucidated the reaction mechanism concerning their structure and interfaces. At a potential of −0.90 V, the FE for CH_3_OH production of Pt_*x*_Zn/C reached its peak at 81.4% (Figure 9c). Additionally, the mixed‐phase Pt_*x*_Zn/C exhibited the lowest Tafel slope value among PtZn/C and Pt_3_Zn/C, indicating more favorable kinetics for CO_2_RR (Figure 9d). Both experimental and theoretical investigations into the mechanistic and kinetic aspects of efficient and selective CO_2_RR over Pt_*x*_Zn suggested that the mixed‐phase material not only facilitated single‐electron transfer to adsorbed CO_2_ but also exhibited improved binding of the intermediate CO_2_^•−^ on its surface. Furthermore, the lower bond energy between the mixed‐phase surface and the adsorbed −OCH_3_, compared to the phase‐pure catalysts, contributed to higher CH_3_OH selectivity over Pt_*x*_Zn (Figure 9e).^[^ ^124^ ^]^ Their work underscores that Pt_*x*_Zn/C can achieve not only higher CH_3_OH selectivity but also reduce interference from HER.

#### 3.3.2 Catalysts for Ethanol Generation

The typical catalysts used in CO_2_RR to C_2_H_5_OH can be found in **Table** **2**. A notable observation is the prevalence of copper‐based catalysts. This underscores the potential of Cu‐based materials as electrocatalysts for CO_2_RR, as Cu^+^ species have shown a favorable affinity for the crucial *CO intermediate in C—C coupling.^[^ ^125^ ^]^

**Table 2:** Summary of representative electrocatalysts used for CO_2_ reduction to ethanol since 2020.

Category	Catalyst	Electrolyte	Potential (V versus RHE)	FE [%]	*J* [mA cm^−2^]
SACs	Cu/C‐0.4^[^ ^18^ ^]^	0.1 m KHCO_3_	−0.7	91	1.23
	TWNa) ‐Cu_13.35_ ‐600‐SAC^[^ ^19^ ^]^	0.5 m CsHCO_3_	−1.1	81.9	35.6
	K–F–Cu–CO_2_^[^ ^183^ ^]^	1 m KOH	−0.56	52.9	423
Heterostructure	dCu_2_O/Ag_2.3%_^[^ ^133^ ^]^	4 m KCl	−0.87	40.8	326.4
	Cu/Cu_2_O^[^ ^28^ ^]^	0.1 m KCl	−1.1	41.2	32.55
	Cu(Ag‐20)_20_^[^ ^62^ ^]^	0.1 m KHCO_3_	−1.1	16.5	4.14
	CuAl_2_O_4_/CuO^[^ ^27^ ^]^	1 m KOH	−0.9	41	82
	FeTPP[Cl]‐Cu^[^ ^184^ ^]^	0.1 m KHCO_3_	−0.91	41.2	124
	Hex‐2Cu‐O^[^ ^59^ ^]^	0.1 m KHCO_3_	−1.2	52i)	9.4
	NGQ/Cu‐nrb) ^[^ ^185^ ^]^	1 m KOH	−0.9	52.4	282.1i)
	CuO_*x*_@C^[^ ^136^ ^]^	1 m KOH	−0.68	46	166
	Cu/N_0.14_C^[^ ^24^ ^]^	0.1 m KHCO_3_	−1.1	51	14.4
	N–C/Cu^[^ ^135^ ^]^	0.2 m KHCO_3_	−3.67	52	156
	Cu–N–G^[^ ^23^ ^]^	0.1 m KHCO_3_	−0.8	33.1	–
	3D Cu–chitosan‐GDL^[^ ^137^ ^]^	1 m KOH	−0.87	51.4	462.6
	PAF‐PA5‐Ag‐0.8^[^ ^138^ ^]^	0.1 m KHCO_3_	−1.05	55	11
	CALc)‐modified Cu NPs^[^ ^90^ ^]^	1 m H_3_PO_4_ + 3 m KCl	−4.2	12	600j)
Monometallic	Cu^+^/hf‐Cud) ^[^ ^129^ ^]^	0.1 m KCl	−0.8	43	–
	Cu_‐DS_ e) ^[^ ^128^ ^]^	0.1 m KHCO_3_	−1.05	67	100i)
	Nanowrinkled Cu^[^ ^130^ ^]^	0.1 m KCl	−0.9	40	–
Intermetallic alloys & coordination	Cu_3_Ag_1_^[^ ^131^ ^]^	0.5 m KHCO_3_	−0.95	63	25i)
	HMMPf) Cu_5_Zn_8_^[^ ^186^ ^]^	0.1 m KHCO_3_	−0.8	46.6	2.3
	Pd–Cu^[^ ^132^ ^]^ (CORR)	1 m KOH	−0.62	40	277i)
	Cu_3_Sn^[^ ^187^ ^]^	0.1 m KHCO_3_	−1.0	64	10
Metal oxide/sulfides	CuO‐FCg) ^[^ ^188^ ^]^	1 m KOH	−1.0	35.7	127
	Cu_2_S_1−*x*_ HNh) ^[^ ^58^ ^]^	0.5 m KHCO_3_	−0.3	73.3	≈2.5
	Cu_2_O NPs^[^ ^21^ ^]^	0.5 m KHCO_3_	−0.6	35.4i)	0.51

a) TWN: thin‐walled nanotubes.

b) NGQ/Cu‐nr: nitrogen‐doped graphene quantum dots on CuO‐derived Cu nanorods.

c) CAL: Cation‐augmenting layer.

d) Cu^+^/hf‐Cu: Cu^+^ sites and high‐facets coexist.

e) Cu_‐DS:_ a defect‐site‐rich Cu structure.

f) HHMP: Hierarchically macroporous–mesoporous.

g) FC: fast cooling rate.

h) Cu_2_S_1−*x*_ HN: Cu_2_S hollow nanocubes with abundant sulfur vacancies.

i) FE or *J* of total alcohols.

j) C_2+_ productivity.

##### Single‐Atom Catalysts (SACs)

**Figure 10:**
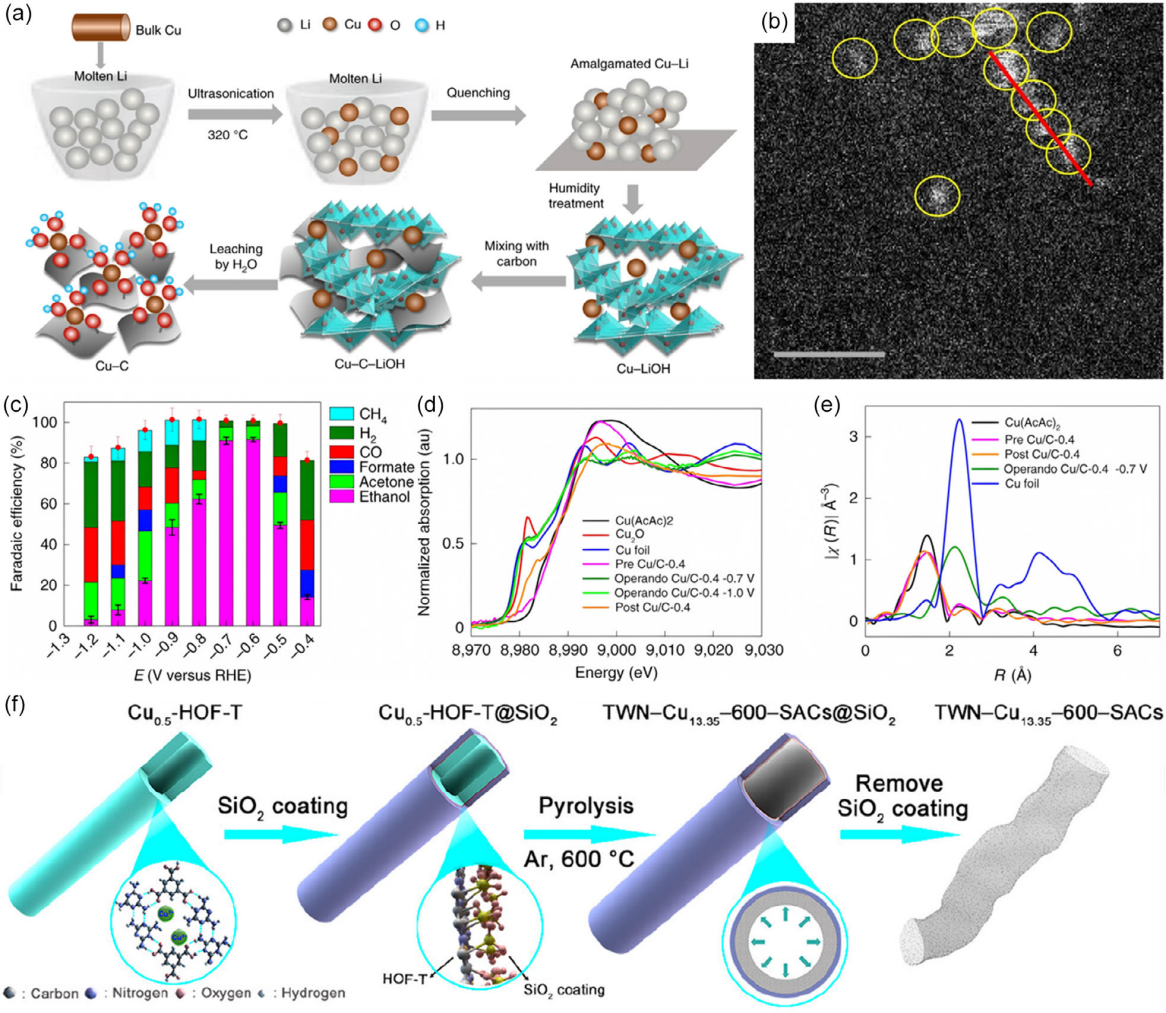
a) Step‐by‐step preparation of the carbon‐supported Cu SA catalyst using an amalgamated Cu–Li method. b) Representative high‐angle‐annular dark‐field and aberration‐corrected STEM images of Cu/C‐0.4 showing the presence of isolated Cu species marked by yellow circles. Scale bar: 1 nm. c) FE and the product distribution at different polarization potentials. d) In situ Cu K‐edge X‐ray absorption near edge structure spectra of pre‐Cu/C‐0.4, Cu/C‐0.4 at −0.7 V versus RHE and −1.0 V versus RHE and post Cu/C‐0.4. e) Fourier transform of *k* ^2^‐weighted *χ* function in *R* space of the catalysts plus Cu(acac)_2_ as a reference. Reproduced with permission.^[^ ^18^ ^]^ Copyright 2020, The Author(s), under exclusive license to Springer Nature Limited. f) Schematic illustration of the fabrication processes of the thin‐walled nanotubes‐shaped TWN‐Cu_13.35_‐600‐SACs catalyst. Reproduced with permission.^[^ ^19^ ^]^ Copyright 2023, American Chemical Society.

Conventional SACs typically lack the ability to catalyze C—C coupling. However, they exhibit efficient performance in facilitating the conversion of CO_2_ into C_2_H_5_OH due to the straightforward nature of single‐atom centers, as indicated by various studies.^[^ ^103^, ^112^, ^126^, ^127^ ^]^ It is worth noting that few studies have discovered the feasibility of SACs to electrocatalytically convert CO_2_‐to‐C_2_H_5_OH. Xu et al.^[^ ^18^ ^]^ have presented findings on a carbon‐supported copper catalyst, prepared with an integrated Cu–Li method (**Figure** **10**a) which demonstrated highly selective CO_2_‐to‐C_2_H_5_OH conversion. All Cu atoms are present as isolated single atoms (SAs), with no NPs detected. Figure 10b details a series of nonbonded Cu SA highlighted by yellow circles. The catalysts achieve an impressive C_2_H_5_OH FE of 91% at −0.7 V versus RHE, with the onset potential as low as −0.4 V for electrocatalytic conversion of CO_2_‐to‐C_2_H_5_OH (Figure 10c). The FE of C_2_H_5_OH is highly dependent on the initial dispersion of Cu atoms and decreases significantly when CuO and large Cu clusters become the predominant species. The dynamic transformation of Cu SA into Cu_*n*_ under CO_2_RR potentials is illustrated in Figure 10d,e, and this transformation is reversible upon reaching the cutoff cell voltage. However, the atomically dispersed Cu atoms were unstable and transformed into Cu_*n*_ clusters (*n* = 3 and 4) under electrochemical conditions. To address this problem, researchers aimed to utilize coordinated sites, such as nitrogen, to bind and stabilize Cu atoms for fabricating Cu–N_*x*_‐coordinated SAs and achieving more stable geometric construction. A silica‐mediated hydrogen‐bonded organic framework‐templated approach has been proposed to preparing ultrahigh‐density Cu–N_3_ SA on TWN nanotubes which favors the conversion of the CO_2_‐to‐C_2_H_5_OH (Figure 10f).^[^ ^19^ ^]^

##### Metals, Alloys, and Metal Oxides/Sulfides

Over the past few decades, the CO_2_RR system has undergone rapid development, leading to the synthesis of various metal‐based electrocatalysts with diverse anionic compositions (metals, alloys, oxides, etc.). These materials typically possess high conductivity, facilitating electron transfer efficiency, and high intrinsic activity, thereby improving the power and energy density of catalyst‐based devices. The catalytic performance of these electrocatalysts is significantly affected by the preparation methods. To optimize catalytic activity, stability, and selectivity, researchers focus on surface engineering, morphology control, and composition manipulation to fine tune the performance of metal‐based electrocatalysts. Consequently, these electrocatalysts are widely studied and commonly applied in the field of CO_2_ conversion to ethanol.

**Figure 11:**
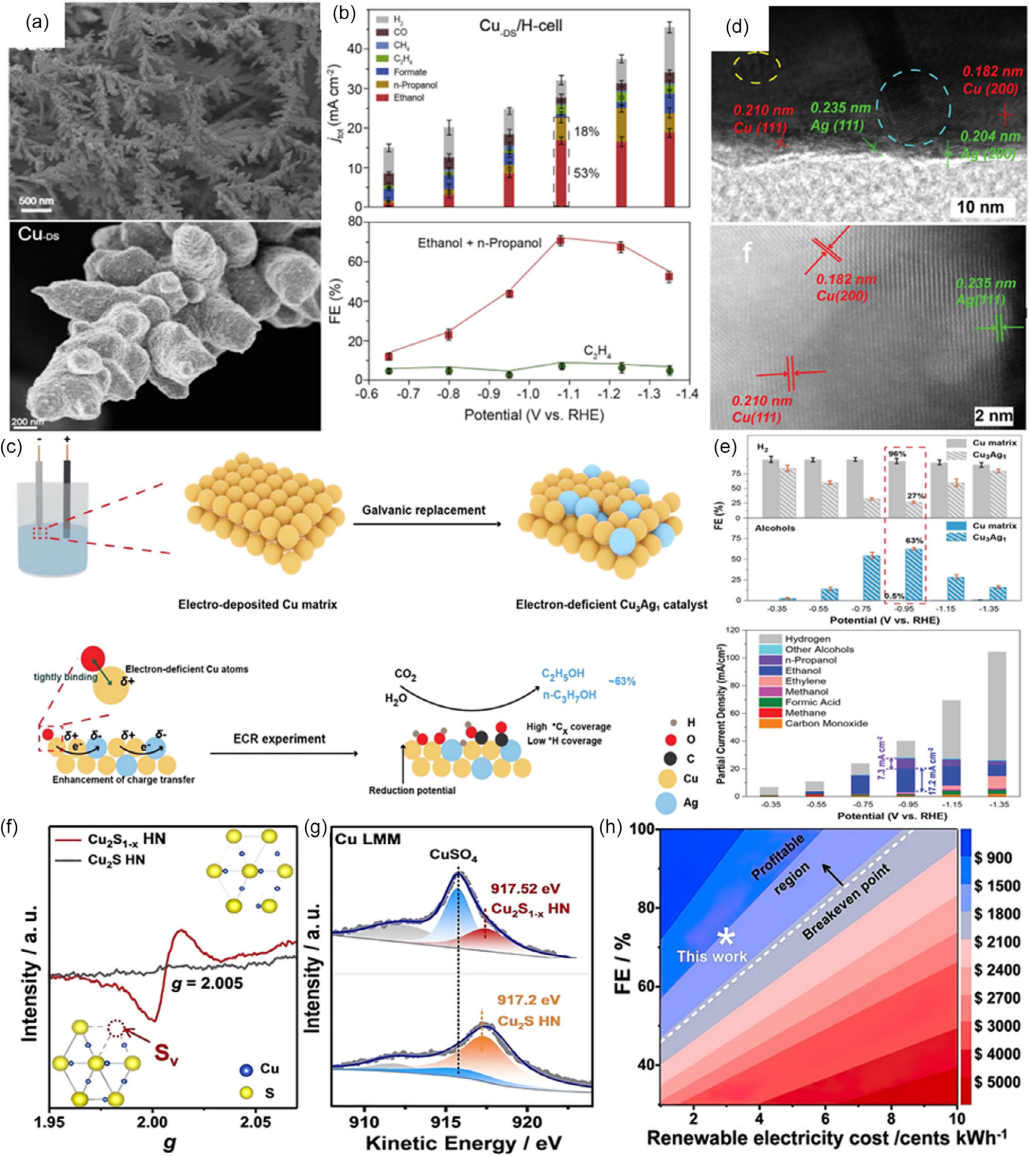
a) SEM and high‐resolution SEM images of the Cu_‐DS_. b) Current densities and product distributions under different potentials (upper panels) and corresponding FEs (low panels) produced by Cu_‐DS_ in H‐Cell. Reproduced with permission.^[^ ^128^ ^]^ Copyright 2020, Elsevier Inc. c) Schematic illustration of the preparation method of the electron‐deficient Cu_3_Ag_1_ catalyst and the electrochemical CO_2_RR process on the Cu_3_Ag_1_ catalyst. d) HRTEM and STEM images of Cu_3_Ag_1_. e) FE_H2_ and FE_alcohols_ for CO_2_RR on Cu matrix and Cu_3_Ag_1_ at different working potentials, and partial current densities for different products on Cu_3_Ag_1_ catalyst at different working potentials. Reproduced with permission.^[^ ^131^ ^]^ Copyright 2020, Wiley‐VCH. f) EPR spectra and g) Cu Auger L‐inner level‐M‐inner level‐M‐inner level electron transition spectra of Cu_2_S_1−*x*_ hollow nanocubes (HN) and Cu_2_S HN. h) Technoeconomic analysis on Cu_2_S_1−*x*_ HN for C_2_H_5_OH generation. Reproduced with permission.^[^ ^58^ ^]^ Copyright 2022, Wiley‐VCH.

The most prevalent strategy is to enrich the defect sites of the catalyst. For example, Gu et al.^[^ ^128^ ^]^ implemented a systematic approach to synthesize a Cu catalyst with a CO‐enriched environment, fostering the development of defect‐rich sites that exhibit optimal CO adsorption capabilities (**Figure** **11**a). This catalyst demonstrated an impressive CO_2_ conversion into C_2+_ alcohols with FE of ≈70% and achieved high current densities surpassing 100 mA cm^−2^ (Figure 11b). Throughout the electrochemical CO_2_ reduction process, the presence of these defect‐rich sites facilitated a heightened surface density of adsorbed ∗CO intermediates, thereby allowing for the fine tuning of CO_2_ electroreduction pathways toward the synthesis of C_2+_ alcohols. Besides, high‐facet atomic arrangements also led to a favorable reaction energy barrier toward C_2_H_5_OH.^[^ ^129^, ^130^ ^]^ The generally low ethanol selectivity has been suggested due to the unfavorable adsorption of several key intermediates (e.g., *CHCHOH, *CH_2_CHOH, *CH_3_CHOH) in the C_2_H_5_OH formation pathway on Cu surfaces. Heteroatomic doping (e.g., Ag, Sn, Zn, Pd) is a powerful strategy to tune the intermediate adsorption capability to improve ethanol selectivity. Lv and colleagues developed a silver‐doped copper matrix catalyst (designated as Cu_3_Ag_1_), using a combination of Cu electrodeposition and subsequent galvanic replacement (Figure 11c).^[^ ^131^ ^]^ The Cu_3_Ag_1_ catalyst, characterized by interphase electron transfer from Cu to Ag, is identified as an electron‐deficient structure serving as the catalytic center. High‐resolution transmission electron microscopy (HRTEM) and scanning transmission electron microscopy (STEM) images reveal the presence of Cu (200) and Cu (111) planes in Cu_3_Ag_1_ (Figure 11d). The calculated lattice constant was ≈0.364 nm, slightly higher than that of the Cu matrix (around 0.354 nm). This difference was attributed to the presence of Ag dopants. Electrochemical experiments confirmed that the adsorption‐tuned Cu_3_Ag_1_ catalyst achieved a total FE of 63% for alcohol production (Figure 11e) with an alcohol partial current density of 25 mA cm^−2^ at −0.95 V versus. RHE. Recently, the secondary metal dopant in bimetallic Cu catalyst can modulate the adsorbed H species and promote alcohol production over ethylene, which confirmed that a loss of adsorbed hydrogen might account for the loss of alcohol selectivity at high productivity.^[^ ^132^ ^]^

Rationally designing abundant and stable Cu^δ+^ active sites is an efficient way to enhance the selectivity and energy efficiency of ethanol over Cu‐based electrocatalysts. Guo et al.^[^ ^58^ ^]^ intentionally synthesized a Cu_2_S_1−*x*_ catalyst rich in Cu^δ+^ (0 < *δ* < 1) species, which exhibited an exceptionally low overpotential of 0.19 V for C_2_H_5_OH production. Impressively, this system exhibited outstanding C_2_H_5_OH selectivity of 86.9% and FE of 73.3%, while also displaying long‐term stability of Cu^δ+^. Computational and in situ spectroscopic analyses unveiled that the abundant Cu^δ+^ sites display electron‐donating ability, leading to a reduction in the reaction barrier during the potential‐determining C—C coupling step (Figure 11f,g). The remarkable attributes of the catalyst, including its ultralow reaction potential and exclusive selectivity for alcohols, have the potential to reduce the overall cost of CO_2_RR and downstream separation processes, providing economic advantages for eventual commercialization (Figure 11h).

##### Heterostructures

**Figure 12:**
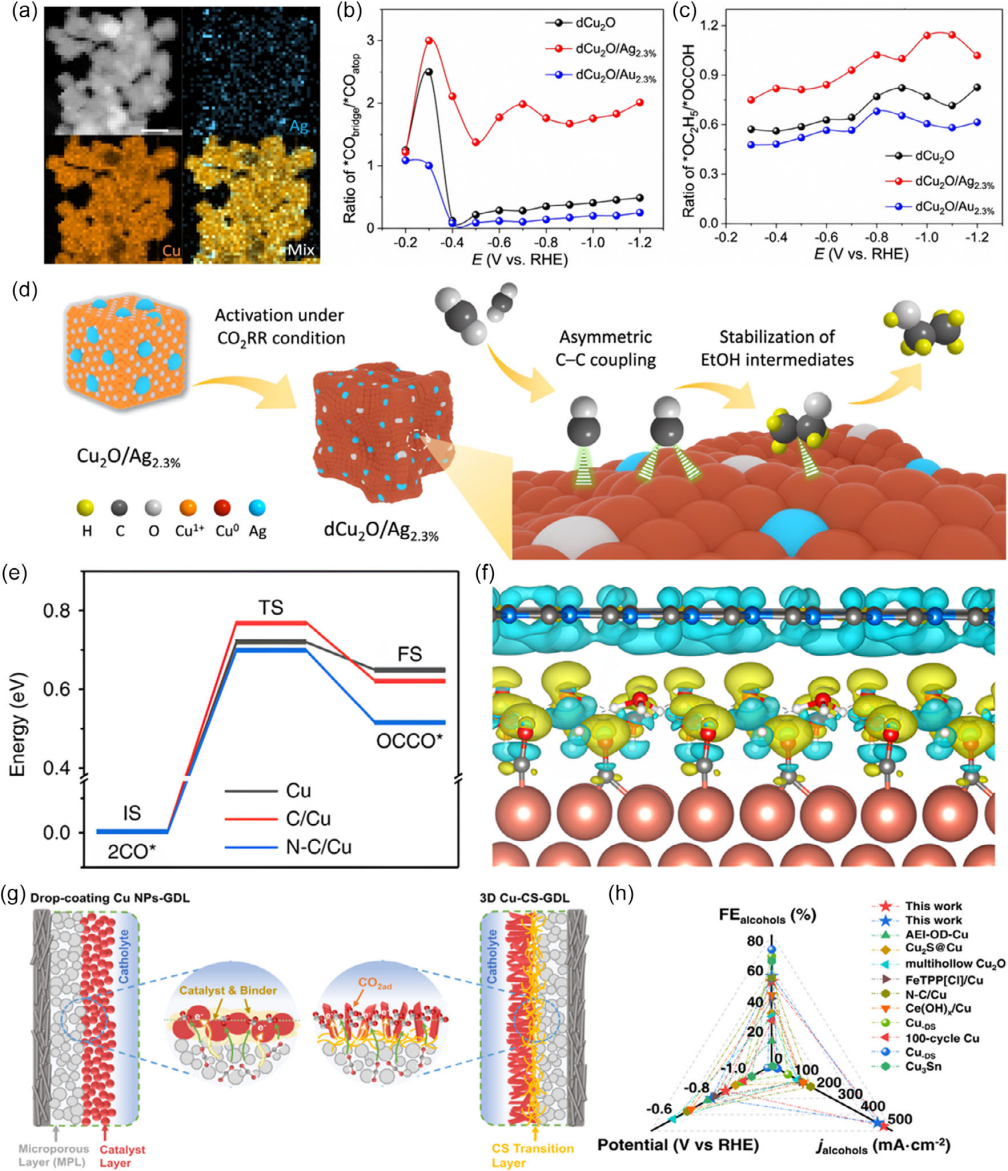
a) Energy‐dispersive X‐ray spectroscopy elemental mapping images of Cu_2_O/Ag_2.3%_. Potential dependence of ratio of b) *CO_bridge_/*CO_atop_ and c) *OC_2_H_5_/*OCCOH obtained for dCu_2_O, dCu_2_O/Ag_2.3%_ and dCu_2_O/Au_2.3%_. d) Schematic for boosted C_2_H_5_OH generation over dCu_2_O/Ag_2.3%_. Reproduced with permission.^[^ ^133^ ^]^ Copyright 2022, The Author(s). e) Energy profiles for initial states (ISs), TSs, and final states of CO dimerization on Cu, C/Cu, and N‐C/Cu, respectively. f) Electron density difference plots for N‐C/Cu. Reproduced with permission.^[^ ^135^ ^]^ Copyright 2020, The Author(s), under exclusive license to Springer Nature Limited. g) Structure of the GDE prepared via the conventional drop‐coating method and 3D Cu–CS‐GDL electrode. h) Comparison of FE of C_2+_ alcohols, the partial current density of C_2+_ alcohols (*J*_alcohols_), and applied potential over 3D Cu–CS‐GDL electrode and typical Cu‐based catalysts reported. Reproduced with permission.^[^ ^137^ ^]^ Copyright 2023, The Author(s).

To boost C_2_H_5_OH production, research interest has concentrated on optimal design of Cu‐based heterostructural catalysts. By combining diverse materials, different heterostructures can be formed, enabling the conversion of CO_2_ to C_2_H_5_OH. These heterostructures can be categorized as inorganic compound‐based, multimetallic composition, and carbon support‐based materials.^[^ ^117^ ^]^ To steer the selectivity toward ethanol, the focus has been on the tuning of the binding strength of reaction intermediates on Cu and lowering the overpotential for C—C coupling via doping using elements such as silver and through the creation of grain boundaries and vacancies. For instance, Wang et al.^[^ ^133^ ^]^ reported a new Ag‐modified copper–oxide catalyst (dCu_2_O/Ag_2.3%_, **Figure** **12**a) that exhibits a significant FE of 40.8% and an energy efficiency of 22.3% for C_2_H_5_OH production. The catalyst achieves selective CO_2_–to–C_2_H_5_OH conversion, yielding a partial current density of 326.4 mA cm^−2^ obtained at −0.87 V versus RHE. The moderate coordination numbers and optimal oxidation for Cu surface in dCu_2_O/Ag_2.3%_ result in tailored *CO configuration (Figure 12b). Therefore, the C—C coupling on dCu_2_O/Ag_2.3%_ could be initiated through asymmetry between *CO and *CHO (or *COH). This asymmetric C—C coupling exhibits a lower energy barrier compared to *CO dimerization, thereby promoting increased C_2_H_5_OH production. The resultant unbalanced coordination environment, induced by the asymmetric C—C coupling, disrupts the coordination sites for C_2_H_4_ intermediates.^[^ ^62^ ^]^ Consequently, the key *OC_2_H_5_ intermediates for C_2_H_5_OH production exhibit greater stability on dCu_2_O/Ag_2.3_ (Figure 12c,d).

Another noteworthy way to enhance C_2_H_5_OH production is the suppression of deoxygenation by coating Cu catalysts with carbon or nitrogen‐doped carbon.^[^ ^134^ ^]^ Coating a nitrogen‐doped carbon (N–C) layer on a Cu has the potential to promote C—C coupling and suppress the breaking of the C—O bond in HOCCH*, thereby promoting ethanol selectivity in CO_2_RR (Figure 12e,f).^[^ ^135^ ^]^ Similarly, Zang et al.^[^ ^136^ ^]^ found that carbon coating can efficiently promote the C—C coupling step and tune the key intermediate *HOCCH to go through the hydrogenation pathway toward C_2_H_5_OH production. In addition to optimizing and regulating the catalytic materials themselves, a 3D Cu‐chitosan (CS)‐GDL electrode has been proposed to promote CO_2_RR performance, in which the CS can act as a “transition layer” between the catalyst and GDL (Figure 12g).^[^ ^137^ ^]^ By systematically tailoring the transition layer, CO_2_ and charge transport in the electrode are optimized to obtain high C_2_H_5_OH productivity (Figure 12h), which represents a novel example of designing efficient GDEs for electrocatalytic CO_2_RR.

Apart from Cu‐based materials, a hydroxypillar[5]arene‐extended porous polymer‐confined silver catalyst (PAF‐PA5‐Ag‐0.8) has been designed for CO_2_ conversion, which demonstrated outstanding activity in promoting the transformation of CO_2_ into C_2_H_5_OH.^[^ ^138^ ^]^ Qin and co‐workers highlight that the enhanced adsorption strength of CO* on PAF‐PA5‐Ag‐0.8, in comparison to commercial Ag NPs, is advantageous for C—C coupling, consequently leading to the selective formation of C_2_H_5_OH.

#### 3.3.3 Catalysts for Propanol Generation

**Table** **3** shows that Cu‐based catalysts are the sole materials investigated for the electrochemical CO_2_RR to C_3_H_7_OH in recent years. C_3_H_7_OH exhibits the highest energy density (27 MJ L^−1^) among the C_1_–C_3_ alcohols, closely comparable to that of gasoline.^[^ ^139^ ^]^ However, the research focus on CO_2_RR to C_3_H_7_OH is considerably less pronounced compared to the emphasis on CO_2_RR to lighter alcohols.

**Table 3:** Summary of electrocatalysts used for CO_2_ reduction to propanol since 2020.

Category	Catalyst	Electrolyte	Potential (V versus RHE)	FE (%)	*J* [mA cm^−2^]
Heterostructure	CAL‐modified Cu NPs^[^ ^90^ ^]^	1 m H_3_PO_4_ + 3 m KCl	4.2	4	600f)
	NGQ/Cu‐nra) ^[^ ^185^ ^]^	1 m KOH	−0.7	27.2	302.4
	CuSx‐DSVb) ^[^ ^140^ ^]^	0.1 m KHCO_3_	−1.05	15.4	3.1
	3D Ni‐SAGc) and Multihollow Cu_2_O^[^ ^85^ ^]^	1 m KOH	–	30.2	12.8
	(Cu_2_O@)_2_Cu_2_O YSNPsd) ^[^ ^20^ ^]^ (CORR)	1 m KOH	–	22.22	50
	Pd–Cu^[^ ^25^ ^]^ (CORR)	1 m KOH	−0.68	46.6	38
	Ag–Ru–Cu^[^ ^26^ ^]^ (CORR)	1 m KOH	−2.75	36	111
Intermetallic alloys and metal oxides	Pd_9_Cu_91_^[^ ^63^ ^]^	0.5 m KHCO_3_	−0.65	13.7	1.15
	CuAg_5%_N_20h_^[^ ^145^ ^]^ (CORR)	1 m KOH	–	45	150
	CuOD‐Cue) ^[^ ^144^ ^]^	1 m KHCO_3_	−0.94	17.9	8.51

a) NGQ/Cu‐nr: graphene quantum dots on CuO‐derived Cu nanorods.

b) DSV: double‐sulfur vacancy.

c) SAG: single‐atom nickel.

d) YSNP: yolk‐shell NPs.

e) CuOD‐Cu: CuO‐derived Cu.

f) C_2+_ productivity.

##### Heterostructure

**Figure 13:**
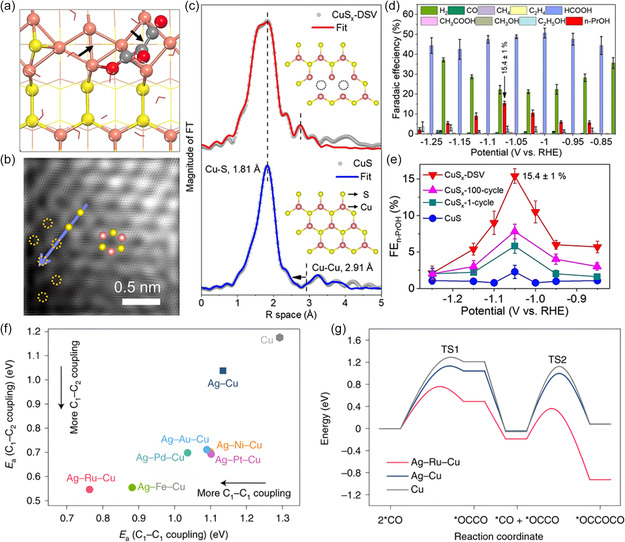
a) Top views of the optimized OCCOCO* intermediate configurations on (100) surface of CuS_*x*_‐DSV. b) High‐angle annular dark‐field‐STEM image of CuS_*x*_‐DSV. c) Fourier‐transform *k* ^2^ *χ*(*k*) of CuS (lower panel) and CuS_*x*_‐DSV (upper panel). d) CO_2_RR product distribution using CuS_*x*_‐DSV catalysts in H‐cells. e) FE of C_3_H_7_OH on the four catalysts at different applied potentials. Reproduced with permission.^[^ ^140^ ^]^ Copyright 2021, The Authors. f) The calculated activation energy (*E*_a_) for C_1_–C_1_ and C_1_–C_2_ coupling on screened Ag–X–Cu, where X is Au, Pd, Pt, Ni, Fe, and Ru. g) Reaction coordinate diagram for C_1_–C_1_ and C_1_–C_2_ coupling on Ag–Ru–Cu, Ag–Cu, and Cu catalyst systems. TS1 and TS2 denote the TS of C_1_–C_1_ and C_1_–C_2_ coupling, that is, *CO–*CO and *CO–*OCCO, respectively. Reproduced with permission.^[^ ^26^ ^]^ Copyright 2022, The Authors.

Catalysts based on heterostructures have been extensively researched for electrocatalytic C_3_H_7_OH production, demonstrating commendable performance. Acquiring C_3_H_7_OH poses a significant challenge due to the intricate process involved in the formation of C_3_, which necessitates the stabilization of *C_2_ intermediates and the subsequent coupling of C_1_–C_2_. Peng et al.^[^ ^140^ ^]^ engineered a catalyst featuring a significant number of sulfur vacancies surrounding the Cu centers (**Figure** **13**a–c). This research revealed that hexagonal Cu sulfide exhibits enhanced electrocatalytic efficiency in the presence of double‐sulfur vacancies, by promoting the stabilization of both CO* and OCCO* dimers. Additionally, this facilitates the coupling of CO and OCCO to form C_3_ species, a process not achievable on CuS when it possesses either a single‐sulfur vacancy or none. The double‐sulfur vacancy on CuS (CuS_*x*_‐double‐sulfur vacancy (DSV)) catalyst exhibited an improved C_3_H_7_OH selectivity, with a peak FE_PrOH_ of 15.4 ± 1% and a corresponding partial current density (*J*_PrOH_) of 3.1 ± 0.2 mA·cm^−2^ at −1.05 V versus RHE (Figure 13d,e).^[^ ^85^, ^141^, ^142^ ^]^ Generally, the carbon loss in CO_2_RR is primarily attributed to the formation of carbonate. Interestingly, a tandem CO_2_RR system involving a two‐step process, with CO_2_RR to CO followed by CORR, has the potential to decrease carbon loss to carbonate in either step. Numerous researchers have been focusing on the design of stable and cost‐effective catalysts with high selectivity toward the electrochemical conversion of CO into C_2+_ products. Multimetallic heterostructure electrocatalysts have demonstrated the ability to achieve impressive catalytic activity and selectivity, all while ensuring stability over prolonged periods. Recent research findings indicate that the introduction of metal‐doped Cu materials has a positive impact on CO adsorption, leading to the stabilization of C_2_ intermediates. This, in turn, facilitates the coupling of C—C and C—C_2_, ultimately enhancing and improving the formation of C_3_ products (Figure 13f,g).^[^ ^26^ ^]^

##### Intermetallic Alloys and Metal Oxide Catalysts

**Figure 14:**
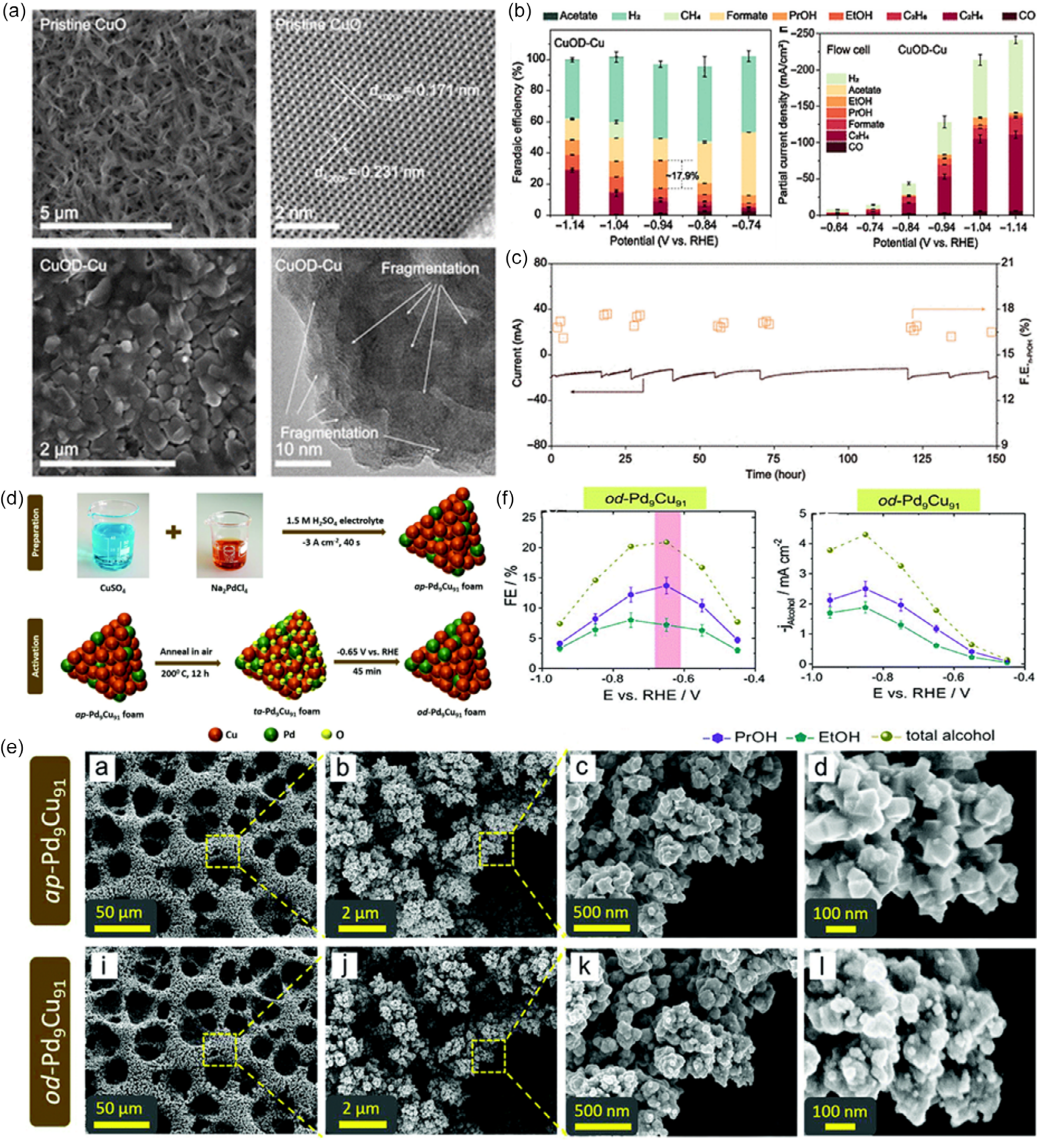
a) SEM images and HRTEM images of pristine CuO and CuOD‐Cu. b) FE of products on CuOD‐Cu and Cu_2_OD‐Cu during CO_2_RR in H‐cell. c) Long‐term stability measurement of CuOD‐Cu. Reproduced with permission.^[^ ^144^ ^]^ Copyright 2023, The Authors. d) Scheme illustrating individual preparation and activation steps of the Pd_9_Cu_91_ catalyst. e) Identical location SEM analysis of the as‐prepared (ap) alloy foam and oxide‐derived (od) alloy foam catalyst depending on the processing stage. f) Alcohol production efficiencies were derived from the electrolysis carried out over the od‐Pd_9_Cu_91_ sample and partial current densities of multicarbon alcohols. Reproduced with permission.^[^ ^63^ ^]^ Copyright 2023, Royal Society of Chemistry.

Intermetallic alloys and metal oxide catalysts have also demonstrated excellent performance in the electrocatalytic conversion of CO_2_ to C_3_H_7_OH. Cu stands out as a unique catalyst capable of electrocatalyzing CO_2_ into valuable fuels and chemicals, especially high‐value C_2+_ products.^[^ ^143^ ^]^ Among Cu‐based materials, oxide‐derived copper has attracted considerable attention for its prowess in C_2+_ production due to its high electrocatalytic activity, straightforward preparation process, and cost‐effectiveness. It is noteworthy that different copper oxides demonstrate diverse performance concerning the selectivity of the final products. This highlights the pressing need for research and development in catalytic materials aimed at enhancing the selectivity of targeted products. Chang et al.^[^ ^144^ ^]^ illustrated that CuO‐derived Cu (CuOD‐Cu) exhibits a more abundant population of undercoordinated Cu sites and an increased density of Cu atoms on its surface compared to the Cu_2_O‐derived Cu (Cu_2_OD‐Cu) during the oxygen removal reconstruction process as depicted in **Figure** **14**a. The outstanding and stable generation of C_3_H_7_OH at low reduction potential is attained on CuOD‐Cu (Figure 14b,c), while formate is the primary product on Cu_2_OD‐Cu.^[^ ^144^ ^]^

Furthermore, researchers discovered that introducing a second‐catalyst component to the activated Cu (C—C coupler, alcohol producer) can further enhance product selectivity toward multicarbon alcohols. Motiar et al.^[^ ^63^ ^]^ introduced an innovative foam‐type electrocatalyst with a high surface area designed for the CO_2_RR. This was achieved through the electrodeposition of a binary PdCu alloy foam, followed by a thermal annealing process that transforms it into its oxidized state (Figure 14d). Subsequent reduction of the oxidized precursors to the metallic state induces significant morphological changes in the Pd_9_Cu_91_ (nominal bulk composition of 9 at% Pd and 91 at% Cu) foam at the nanometer scale as depicted in Figure 14e. The distinctive structure of Pd_9_Cu_91_ facilitates the release of CO from the catalyst while simultaneously incorporating the necessary C—C coupling element. This contributes to a high selectivity toward the formation of C_3_H_7_OH (FE_PrOH_ = 13.7%, *J*_PrOH_ = 1.15 mA cm^−2^) at relatively modest overpotentials (−0.65 V versus RHE) (Figure 14f). Similarly, Hong and co‐workers^[^ ^145^ ^]^ presented an innovative Cu‐based catalyst known as CuAg_5%_N_20h_ (a sample obtained with 5% of Ag in the galvanic exchange reaction solution and after nitridation during 20 h), which demonstrates remarkable selectivity, achieving a record‐high 45% formation of C_3_H_5_OH. This notable performance is attributed to the presence of Ag doping within the catalyst structure. While direct electrocatalytic CORR proves effective in promoting C_3_H_7_OH generation, the practicality of utilizing CO as a feedstock for the industrial or decentralized production of multicarbon products is hindered by the risks associated with high‐pressure storage of CO and high maintenance costs of the equipment. Thus, the tandem CO_2_RR system has attracted considerable interest in recent years as a solution to mitigate the challenges associated with high‐pressure CO storage.^[^ ^85^ ^]^ Nevertheless, cost concerns pose significant implications in the design of electrocatalysts loaded with precious metals, such as Ag and Au, despite their remarkable performance in several applications.^[^ ^146^, ^147^ ^]^

## 4 Critical Conditions Affecting CO_2_RR

### 4.1 Reaction Pressure

The products of CO_2_RR experiments are influenced by various factors, encompassing the catalyst type, electrolyte solution, CO_2_ concentration, reaction pressure, reaction temperature, and pH value. These variables collectively impact the pathways of CO_2_ conversion. The challenge arises from the low solubility of CO_2_ in aqueous solvents, hindering sustained high‐current‐density operation and fostering competition with the HER. This competition diminishes the availability of CO_2_ at the cathode in the H‐Cell. Addressing this challenge can be effectively accomplished by elevating the system's pressure. Henry's law elucidates that increasing pressure enhances CO_2_ solubility while maintaining an aqueous solvent environment. Consequently, elevated CO_2_ solubility correlates with increased CO_2_ reduction rate, current density, and overall reaction efficiency. However, employing extremely high‐pressure conditions for C_2_H_5_OH production may pose potential risks and substantially higher production costs. Consequently, the investigation of pressure's impact on alcohol generation remains largely unexplored.^[^ ^148^ ^]^ The selective production of alcohols by establishing appropriate pressure conditions represents a promising avenue for investigation and warrants further exploration.

### 4.2 Reaction Temperature

The significance of reaction temperature in electrochemistry, particularly in CO_2_RR, is often overlooked. In recent studies, various research groups have explored the impact of temperature on the FE of different products during CO_2_RR. The influence of temperature on CH_3_OH production was investigated by Hossain et al.^[^ ^149^ ^]^ which showed opposite product distribution when the temperature ranged from 20 to 50 °C. The study demonstrated that H_2_ formation exhibited a positive correlation with temperature, while CH_3_OH, CO, and HCOOH showed the opposite trend. The highest CH_3_OH selectivity, with FE exceeding 36%, was observed at − 0.6 V (versus RHE) and 20 °C. Similarly, Vos and co‐workers^[^ ^150^ ^]^ identified two distinct temperature regimes. In the range of 18–48 °C, C_2+_ products exhibited higher FE, whereas CH_4_ and HCOOH selectivity decreased, and hydrogen selectivity remained approximately constant. From 48 to 70 °C, a HER‐dominated process was found, leading to a decrease in the production of alcohols. This phenomenon is attributed to the fact that low temperatures can reduce the CO_2_ diffusion coefficient, reaction kinetics, and ionic conductivity while increasing CO_2_ solubility. Consequently, selecting an appropriate temperature range becomes crucial based on specific research conditions and objectives. This ensures an optimal balance between CO_2_ solubility and diffusion coefficient.

While the enhancement of CO_2_ reduction kinetics is observed with rising temperature, alkaline electrolyzers exhibit diminished CO_2_ availability as temperature increases, ultimately hindering reaction productivity.^[^ ^151^ ^]^ There appears to be a correlation between higher operating temperatures and increased currents. Nevertheless, this relationship is intricate, given that higher temperatures result in lower CO_2_ solubility, accompanied by changes in pH. Yet they also lead to higher diffusion coefficients and reactivity. Further investigation is necessary to fully understand the underlying mechanisms by which temperature influences CO_2_RR.

### 4.3 pH Value of Electrolytes

Another crucial factor to take into account is the impact of pH on CO_2_RR catalytic performance. In general, the equilibrium of CO_2_ hydrolysis shifts toward (bi)carbonates in high pH, reducing the local concentration of CO_2_ and hampering its transport, which is deemed detrimental to electrocatalysis. Studies indicate that CO_2_RR in an acidic environment is more efficient, particularly when there is a high concentration of potassium cations near the electrochemically active sites.^[^ ^90^ ^]^ This efficient process occurs on copper at a pH level below 1, achieving a single‐pass CO_2_ utilization of 77%. This includes a conversion efficiency of 50% toward multicarbon products such as C_2_H_4_, C_2_H_5_OH, and C_3_H_7_OH, at a current density of 1.2 A cm^−2^ and a full‐cell voltage of 4.2 V. Contrastingly, Bagchi and colleagues found that at high pH values, the formation of C_2+_ products was favored over CH_4_.^[^ ^152^ ^]^ They observed that the CH_4_ formation is prone to occur at lower H^+^ concentrations, while the RLS in the formation of C_2+_ products is pH independent.

## 5 Challenges and Future Perspectives

### 5.1 Low CO_2_ Solubility

The sluggish mass transport resulting from the limited solubility of CO_2_ in electrolytes significantly hampers the occurrence of CO_2_RR on the electrode, particularly in the H‐cell.^[^ ^153^, ^154^ ^]^ Enhancing local CO_2_ concentration is advantageous for achieving high current densities through the rational design for GDE and the modification of the gas–liquid–solid interface. However, the phenomenon of the “salting‐out” effect, wherein high current densities lead to decreased CO_2_ solubility with increasing total salt concentration, poses a challenge that requires further exploration. By addressing the challenge of poor CO_2_ solubility in aqueous solvents, researchers have turned to ILs and organic electrolytes with much higher CO_2_ solubility. For instance, solvents like dimethyl sulfoxide exhibit approximately four times higher solubility, while CH_3_CN and dimethylformamide demonstrate approximately four and twenty times higher solubility, respectively, compared to H_2_O or C_2_H_5_OH/CH_3_OH. In these alternative solvents, the concentration of the dissolved water plays a crucial role in manipulating proton availability and, consequently, the FE of the reaction products.^[^ ^155^, ^156^ ^]^ Unfortunately, the economic viability of these alternatives is hindered by their high cost and possible toxicity, making them less conducive to long‐term, large‐scale applications.

### 5.2 Industrial and Economic Feasibility

The primary objective of electrocatalytic CO_2_RR is to achieve industrialization and unlock its economic and social benefits. While extensive research at the laboratory scale has showcased the feasibility and potential of CO_2_RR in alcohol production, the transition to industrial applications necessitates additional criteria and economic analyses, which include high current density (exceeding 200 mA cm^−2^), ensuring a product selectivity over 90% and demonstrating long‐term stability (beyond 1000 h) to access the viability of CO_2_RR on an industrial scale.^[^ ^6^, ^157^ ^]^ To meet these requirements, it is crucial to systematically design and optimize the CO_2_RR system. One effective and feasible approach is to enhance the current density, as it reduces the investment required for CO_2_ reduction technology. The benefits of achieving high current density and low activation overpotential include increased energy efficiency and enhanced market competitiveness. Another critical aspect is the stability and durability of the catalyst, which are pivotal parameters for ensuring long‐term practical implementation, which can not only reduce the maintenance and replacement costs but also minimize associated downtime. However, achieving long‐term durability for electrocatalysts in alcohol production remains a significant challenge, with ongoing debates about the key factors leading to deactivation. Despite prioritizing the optimization of the electrocatalyst performance and notable progress in this regard, high‐performance electrocatalysts still face inevitable challenges.^[^ ^158^, ^159^, ^160^ ^]^

Moreover, the catalyst exhibits a low selectivity, specifically in its conversion to high‐value products. For instance, the reported FE for C_2_H_4_ is below 60%, and the FE for other C_2+_ products, including C_2_H_5_OH and C_3_H_7_OH, is even more diminished.^[^ ^36^ ^]^ This reduced FE imposes significant challenges on the subsequent separation and purification processes for the generated products. The utilization of an aqueous salt electrolyte (KHCO_3_, KOH, K_2_CO_3_) results in low purity and concentration of the liquid products.^[^ ^72^ ^]^ Consequently, costly techniques such as reverse osmosis or electrodialysis become necessary for the separation of these liquid products from a mixture of impurity ions, rendering the CO_2_RR process economically unfeasible. Jouny et al.^[^ ^161^ ^]^ conducted a comprehensive examination of contemporary metrics for CO_2_ reduction and performed an economic assessment to calculate the net present value at the end of the lifecycle for a generic CO_2_ electrolyzer system designed to produce various CO_2_‐reduced products at a daily rate of 100 tons. Their findings revealed that the average production cost for CH_3_OH via CO_2_RR production is $1.4 per kg, significantly exceeding the market price of $0.26 per kg. This results in an average/market price ratio of 547% with a standard deviation/average ratio of 74%.^[^ ^162^ ^]^

### 5.3 Insufficient Understanding of Reaction Mechanisms

Selective CO_2_RR via C—C coupling to C_2+_ alcohols is extremely challenging due to the multiple pathways involved in the reaction process.^[^ ^163^, ^164^ ^]^ Thus, the reaction pathways as well as the kinetic and thermodynamic parameters governing the process have not been uniformly identified. Although tremendous efforts have been made to investigate the CO_2_RR mechanism, numerous aspects at the molecular level remain unclear or even contentious with the underlying mechanisms posing ongoing questions. Theoretically, the activation energy trends of the proton–electron transfer to various intermediates provide insights into the reaction mechanism and RLS for the CO_2_RR. Nevertheless, the electrochemical activation energies of intermediates exhibit notable variations across various studies due to differences in assumptions made during the establishment of electrochemical interfaces. This is the main reason why the reaction mechanism of CO_2_RR to produce many multicarbon products has never been unified. For instance, Chang et al.^[^ ^60^ ^]^ proposed a shared common intermediate, methyl carbonyl, between ethylene and C_3_H_7_OH in the production of C_3_H_7_OH. They conjectured that methyl carbonyl might also serve as the intermediate where the reaction pathway in the CORR bifurcates into C_2_ and C_3_ products. Interestingly, Silva and co‐authors made an opposite conclusion,^[^ ^165^ ^]^ indicating a need for further investigations to achieve a more reliable consensus. A pressing challenge in the field is the imperative need to unravel the intricacies of the CO_2_RR mechanism. Microkinetic modeling techniques are powerful tools to gain a mechanistic understanding of the process without simplifying and limiting assumptions regarding the nature of the rate‐determining steps and the presence of abundant surface intermediates.^[^ ^166^ ^]^ Research advancements in this field would contribute to the understanding of the complex reaction pathways leading to the formation of alcohols and the nature of electrocatalyst active sites and surface chemistry. Solving these critical aspects is essential to substantially advance the design and optimization of novel and more efficient catalysts and unlock the full potential of CO_2_ reduction technologies.

### 5.4 Research on Integrating Electrochemical Process with Other Catalytic Systems

Though remarkable progress that has been made on the electrocatalytic conversion of CO_2_ into low‐carbon alcohols driven by renewable electricity, recycling CO_2_ into energy‐rich long‐chain products of higher energy density and economic value is rarely reported. This is primarily due to the strict reaction conditions and the enigmatic nature of the reaction mechanisms.^[^ ^167^ ^]^ Increasing efforts have been made toward transforming CO_2_ into long‐chain compounds by integrating electrochemical and biological systems. Examples include glucose,^[^ ^168^ ^]^ poly‐3‐hydroxybutyrate,^[^ ^169^ ^]^ and phenylpropenes.^[^ ^170^ ^]^ The production of these compounds in a hybrid electrobiosystem shows great promise and possibility in coupling systems. Developing alternative methods for generating products such as fatty acids using CO_2_ as the primary carbon source continues to pose a significant and unresolved challenge. With increasing demands for upcycling CO_2_ into value‐added products, multisystem coupling strategies can be broadly applied to investigate sustainable development in the future.

## 6 Conclusion

Research enthusiasm for electrocatalytic CO_2_RR to alcohols has reached an unprecedented climax and is increasing due to its ability to produce green fuels and alleviate the greenhouse effect. In this review, we focus on the discussion of electrocatalytic CO_2_RR to alcohols. First, we critically summarize the CO_2_RR reactor design, electrolyte selection, and electrocatalysts for CO_2_RR to CH_3_OH, C_2_H_5_OH, and C_3_H_7_OH. Following this, considering the complex multistep electron and proton transfers involved during CO_2_RR for forming alcohols, we elucidate the main reaction pathways and mechanism for CO_2_RR production of CH_3_OH, C_2_H_5_OH, and C_3_H_7_OH proposed in the literature from the perspective of thermodynamics and kinetics. The formation pathways of CH_3_OH can be divided into the CO route and the formyl route, which have generally been recognized by researchers. It is worth noting that there is still much controversy over the production pathway of C_2_H_5_OH and C_3_H_7_OH. The main difference is that the generation of multicarbon alcohols involves the formation of C—C bonds and the protonation process of multiple carbon atoms. Furthermore, the effects of reaction operating conditions such as pressure, temperature, and pH on the electrochemical CO_2_RR products have been discussed. Selecting the most appropriate reaction conditions is crucial to achieve more efficient production goals in practical applications targeting different product requirements. Finally, key challenges include improving energy efficiency, increasing process selectivity, and reducing system and operating costs. Moving forward, continued research efforts focused on mechanistic investigations and exploration of coupling methods will be instrumental in driving innovation and facilitating the transition toward a sustainable carbon economy.

## Conflict of Interest

The authors declare no conflict of interest.
